# Advancing clinical genomics and precision medicine with GVViZ: FAIR bioinformatics platform for variable gene-disease annotation, visualization, and expression analysis

**DOI:** 10.1186/s40246-021-00336-1

**Published:** 2021-06-26

**Authors:** Zeeshan Ahmed, Eduard Gibert Renart, Saman Zeeshan, XinQi Dong

**Affiliations:** 1 Rutgers Institute for Health, Health Care Policy and Aging Research, Rutgers University, 112 Paterson Street, New Brunswick, NJ USA; 2 Department of Medicine, Robert Wood Johnson Medical School, Rutgers Biomedical and Health Sciences, 125 Paterson Street, New Brunswick, NJ USA; 3 Rutgers Cancer Institute of New Jersey, Rutgers University, 195 Little Albany St, New Brunswick, NJ USA

**Keywords:** Annotation, Disease, Gene, Expression, Heat map, RNA-seq

## Abstract

**Background:**

Genetic disposition is considered critical for identifying subjects at high risk for disease development. Investigating disease-causing and high and low expressed genes can support finding the root causes of uncertainties in patient care. However, independent and timely high-throughput next-generation sequencing data analysis is still a challenge for non-computational biologists and geneticists.

**Results:**

In this manuscript, we present a findable, accessible, interactive, and reusable (FAIR) bioinformatics platform, i.e., GVViZ (visualizing genes with disease-causing variants). GVViZ is a user-friendly, cross-platform, and database application for RNA-seq-driven variable and complex gene-disease data annotation and expression analysis with a dynamic heat map visualization. GVViZ has the potential to find patterns across millions of features and extract actionable information, which can support the early detection of complex disorders and the development of new therapies for personalized patient care. The execution of GVViZ is based on a set of simple instructions that users without a computational background can follow to design and perform customized data analysis. It can assimilate patients’ transcriptomics data with the public, proprietary, and our in-house developed gene-disease databases to query, easily explore, and access information on gene annotation and classified disease phenotypes with greater visibility and customization. To test its performance and understand the clinical and scientific impact of GVViZ, we present GVViZ analysis for different chronic diseases and conditions, including Alzheimer’s disease, arthritis, asthma, diabetes mellitus, heart failure, hypertension, obesity, osteoporosis, and multiple cancer disorders. The results are visualized using GVViZ and can be exported as image (PNF/TIFF) and text (CSV) files that include gene names, Ensembl (ENSG) IDs, quantified abundances, expressed transcript lengths, and annotated oncology and non-oncology diseases.

**Conclusions:**

We emphasize that automated and interactive visualization should be an indispensable component of modern RNA-seq analysis, which is currently not the case. However, experts in clinics and researchers in life sciences can use GVViZ to visualize and interpret the transcriptomics data, making it a powerful tool to study the dynamics of gene expression and regulation. Furthermore, with successful deployment in clinical settings, GVViZ has the potential to enable high-throughput correlations between patient diagnoses based on clinical and transcriptomics data.

**Supplementary Information:**

The online version contains supplementary material available at 10.1186/s40246-021-00336-1.

## Introduction

Over the past few years, genomic sequencing technologies have improved the clinical diagnosis of genetic disorders and continue to expand the potential of basic sciences in developing insights into human genetic variations and their biological consequences. Gene expression analysis is a widely adopted method to identify abnormalities in normal function and physiologic regulation [1]. It supports expression profiling and transcriptomic analyses to identify, measure, and compare genes and transcripts in multiple conditions and in different tissues and individuals. Several recently published studies have shown that gene expression analysis is a proven method for understanding and discovering novel and sensitive biomarkers among several complex disorders. Two major techniques that are currently being used for gene expression analysis are microarrays and RNA sequencing (RNA-seq) [2]. Microarrays are based on traditional microarray platforms for transcriptional profiling that quantify a set of predetermined whole transcriptome sequences [3], while RNA-seq identifies, characterizes, and quantifies differentially modulated transcriptomes [4]. Due to recent advancements in next-generation sequencing (NGS) technologies and the development of new bioinformatics applications, RNA-seq has become the most widely used method for gene expression analysis [5].

Several RNA-seq data pre-processing pipelines have been developed and published and are freely available [4]. Most of the pipelines follow a similar workflow, which starts with quality checking the sequences, trimming barcodes, sorting sequences, removing duplicates, aligning to reference genome and transcriptome, and calculating different metrics. RNA by expectation maximization (RSEM) is a widely applied and proven algorithm for the quantification and identification of differentially expressed genes (DEGs) that aligns sequences to reference de novo transcriptome assemblies [6]. Its outcomes include quantified gene and isoform abundances with transcripts per million (TPM), fragments per kilobase million (FPKM), reads per kilobase of transcript per million mapped reads (RPKM), and mean expressed transcript lengths. These values are mainly used in case-control studies and gene expression analysis, which requiring bioinformatics expertise to understand the processed RNA-seq data complexities, and computational methods and programming languages to interpret, visualize, and report produced analytic results.

Data visualization is considered essential for RNA-seq interpretation, as it bridges the gap between algorithmic approaches and the cognitive skills of users and investigators. Over the past decade, different data visualization tools have emerged. Some are available as commercial packages (e.g., Tableau, Heatmap.me, Hotja, Crazy Egg, Inspectlet), and others include academic open-source code applications (e.g., BEAVR, NOJAH, Heatmap3, Clustergrammer). However, based on our evaluation, most of these tools are slow; sometimes unable to render large RNA-seq datasets; downloadable, but difficult to install and configure; available only with manual data uploading and management; not freely available and require subscriptions (commercial only); and, lastly, not user-friendly but require good knowledge of programming languages and computational skill sets.

Independent and timely high-throughput NGS data analysis is still a challenge for non-computational biologists and geneticists. In this study, we are focused on supporting RNA-seq-driven gene expression data analysis, annotation with relevant diseases, and heat map visualization without requiring a strong computational background from the user. We present GVViZ (visualizing genes with disease-causing variants), a newly developed bioinformatics application for gene-disease data visualization, annotation, and expression analysis with a dynamic heat map visualization. GVViZ is a findable, accessible, interactive, and reusable (FAIR) platform, based on a set of seven simple instructions that will allow users without computational experience (e.g., bench scientists, non-computational biologists, and geneticists) to analyze data, visualize data, and export data to share results.

## Material and methods

**Fig. 1:**
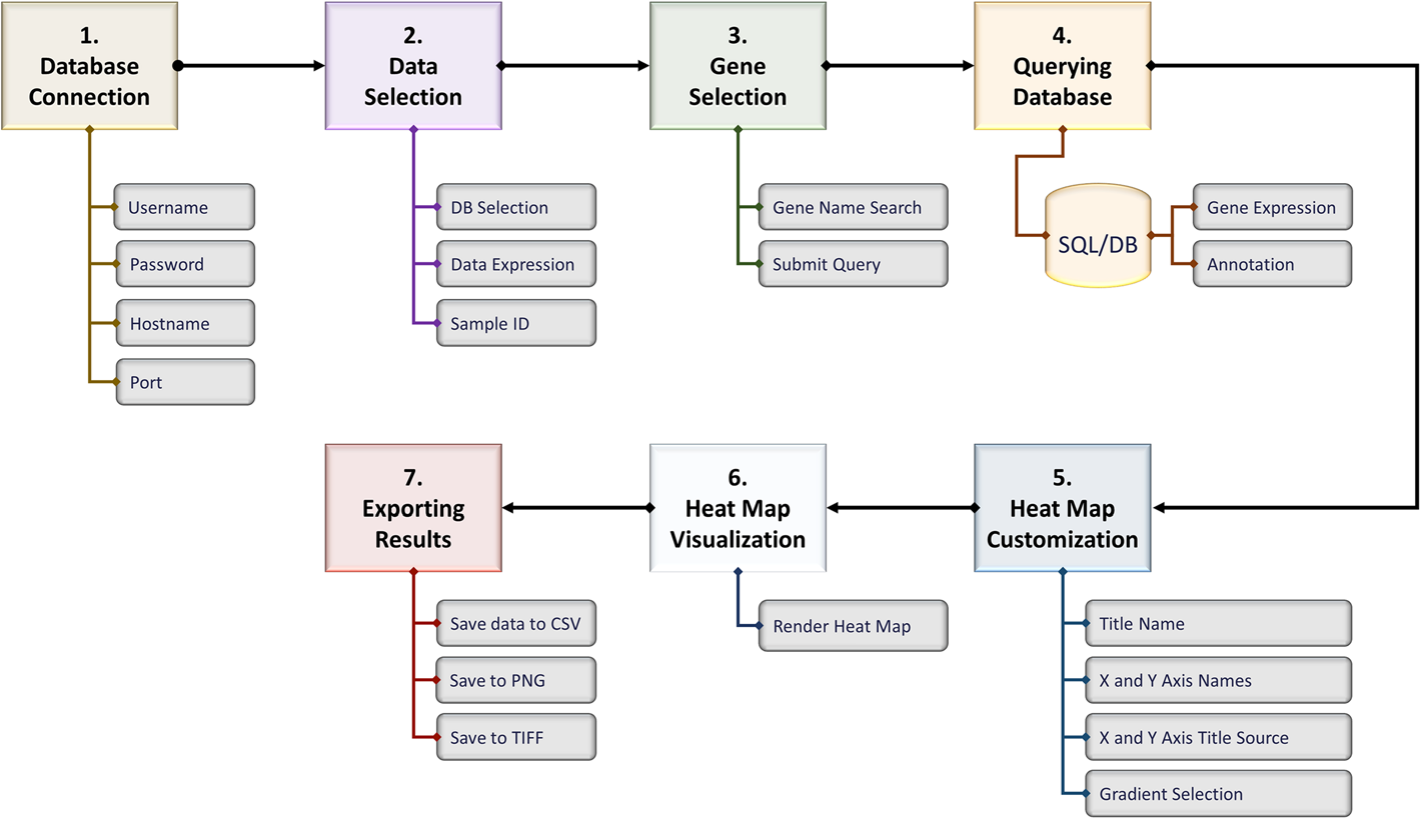
GVViZ workflow design. Overall tasks include the following: (1) database connection, (2) data selection, (3) gene selection, (4) querying database, (5) heat map customization, (6) heat map visualization, and (7) exporting results

**Fig. 2:**
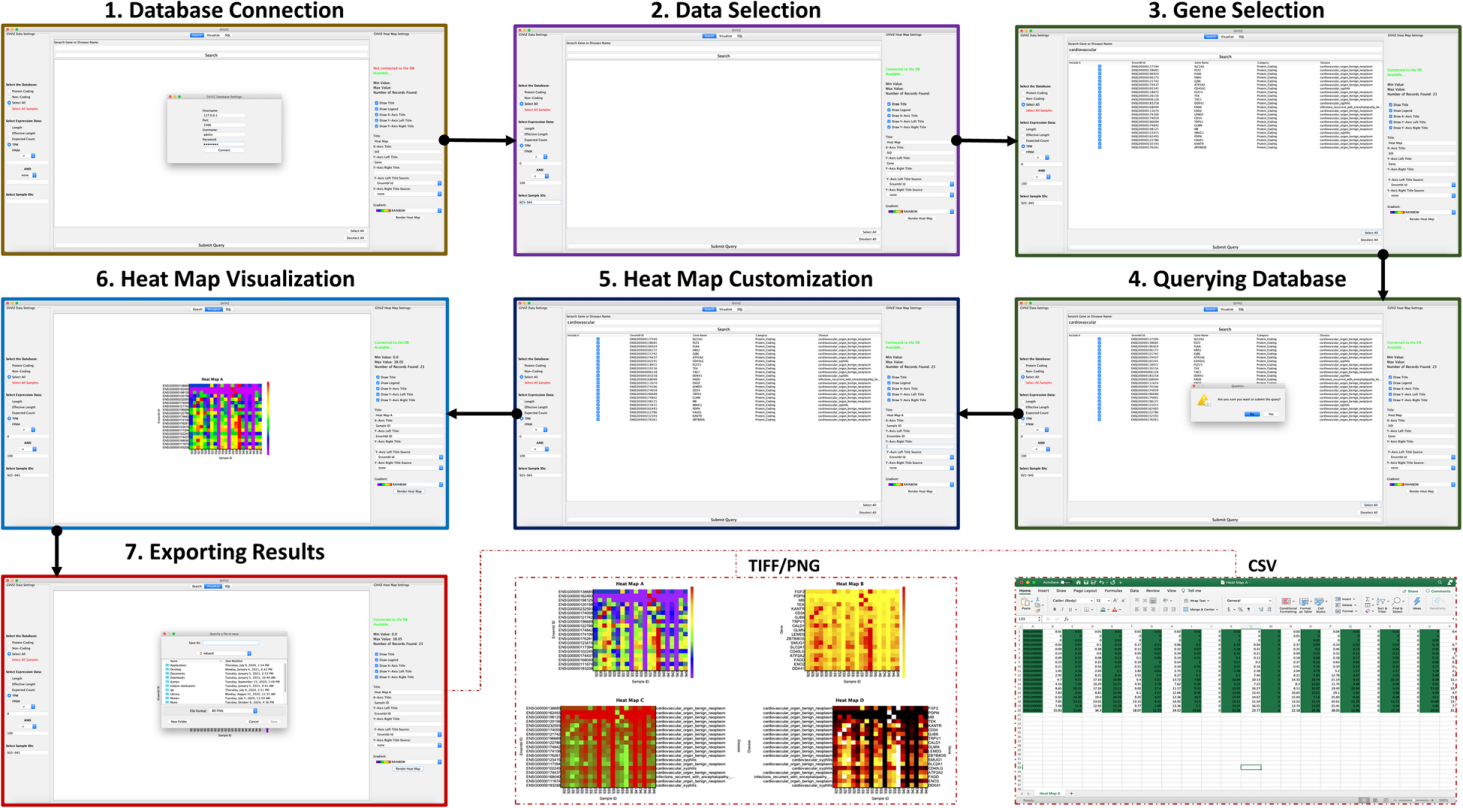
GVViZ graphical user interface. Sequence of screenshots explaining the overall interactive interfaces

GVViZ is a robust bioinformatics, user-friendly, cross-platform, desktop, and database application. Figure 1 explains the workflow and Fig. 2 demonstrates the graphical user interface (GUI) of GVViZ, which includes (1) database connection, (2) data selection, (3) gene selection, (4) querying database, (5) heat map customization, (6) heat map visualization, and (7) exporting of results. The database connection step establishes a link to the SQL server using authenticated user credentials. Data selection allows the user to select among gene types, expression values, and samples. Gene selection offers features to search and select genes and associated diseases for the analysis. Querying database triggers annotation and gene expression analysis based on selected abundances and pruning conditions, samples, genes, and diseases. Heat map visualization provides features to customize and render heat maps, and exporting results allows the user to save outcomes as image (PNG/TIFF) or text (CSV) files.

**Fig. 3:**
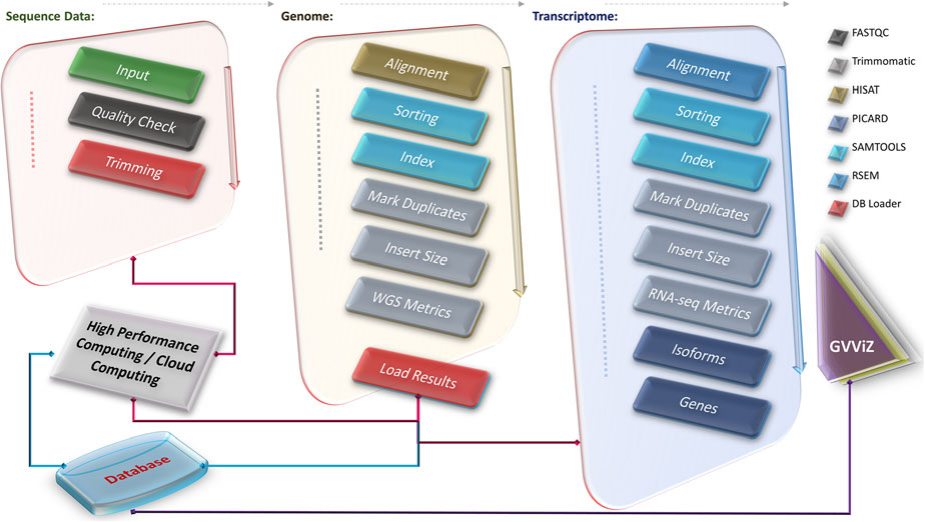
RNA-seq data processing pipeline. We use FastQC for quality checking, Trimmomatic to remove adapters and low-quality sequences, SAMtools to sort and index sequences, MarkDuplicates to remove duplicates, CollectInsertSizeMetrics to compute the size distribution and read orientation of paired-end libraries, HISAT with Bowtie2 to align the sequences to the human reference genome, and RSEM to quantify and identify differentially expressed genes by aligning reads to reference de novo transcriptome assemblies. Furthermore, our RNA-seq pipeline utilizes an in-house developed software application to automatically parse the outcome files of the pipelines and upload the results into a modeled relational database, which are then used by GVViZ for data annotation, analysis, and visualization

GVViZ simplifies the process of gene expression data analysis, visualization, and exploration of results by using an SQL database, which is divided into two relations: gene expression data and gene-disease annotation. The results of the RNA-seq data processing pipeline (Fig. 3) are automatically parsed and uploaded into gene expression data, which includes TPM, FPKM, expressed transcript lengths, and counts for each of the samples. TPM values are proven to be more accurate measures of the true abundance of RNA molecules from given genes, and TPM counts are more consistent across libraries. Therefore, they potentially allow a more stable statistical analysis. The goal is to perform a gene expression study, where the gene-level TPM estimates, representing the overall transcriptional output of each gene, are compared between conditions.

The gene-disease annotation relation is populated with published literature-based annotated gene-disease data, collected from different clinical and genomics databases [7], and categorized among gene name/ID, Ensembl ID, category (protein-coding or non-protein-coding, both protein-coding and non-protein-coding, or all available genes in processed RNA-seq samples), and relevant disease (Fig. 1). Our RNA-seq pipeline (Fig. 3) implements FastQC for quality checking [8], Trimmomatic to remove adapters and low-quality sequences [9], SAMtools to sort and index sequences [10], MarkDuplicates to remove duplicates [11], CollectInsertSizeMetrics to compute the size distribution and read orientation of paired-end libraries, and HISAT with Bowtie2 to align the sequences to the human reference genome (hg38) [12, 13]. RSEM is then applied for quantification and identification of differentially expressed genes by aligning the reads to reference de novo transcriptome assemblies [6]. As the final step, our RNA-seq pipeline utilizes an in-house developed scientific software application to efficiently extract and parse information from most of the outcome files (e.g., QC metrics, genes, isoforms) and transfer and load into a modeled relational database. The results based on genes are automatically linked to GVViZ for further annotation, expression analysis, and heat map visualization. Our RNA-seq pipeline starts with mainly the input of short read-based FASTQ files, preferably produced by the Illumina sequencing technology. We recommend the use of paired-end reads, but our pipeline can also work at single-end reads. Users can customize this pipeline based on their needs and can start from any point they would, e.g., instead of starting with FASTQ files, if they have already created SAM/BAM files, they can start directly using HISAT with Bowtie2, and RSEM.

Once GVViZ is successfully connected to the SQL database server, it allows users to design the analysis, select single and multiple sample cohorts, and customize visualization. GVViZ provides SQL-based features to search and select genes and their associated diseases to support gene-disease data annotation. Next, users can select the appropriate gene category (coding, non-coding, both, or all available in samples used for analysis) for the designated analysis. Users need to define the criteria by choosing the right abundance type, setting desired minimum and maximum values, and selecting applicable analytic conditions. Users can select control and diseased samples from the main cohort by picking individual samples and define the range among one or multiple cohorts. GVViZ provides features to customize data visualization, which include titles (header, right y-axis, left y-axis, and x-axis), color schemes (28 gradients), selection and positioning of values (right y-axis and left y-axis), and rendering of heat maps. Finally, users can visualize the results within the GVViZ data visualization panel, as well as export in image (TIFF and PNG) and text (CSV) formats (Fig. 2).

To advance our clinical genomics and precision medicine study, we modeled and implemented an annotated disease-gene-variants database that includes but is not limited to data collected from several genomics databases worldwide [7], including PAS [14, 15], ClinVar [16], GeneCards [17], MalaCard [18], DISEASES [19], HGMD [20], Disease Ontology [21], DiseaseEnhancer [22], DisGeNET [23], eDGAR [24], GTR [25], OMIM [26], miR2Disease [27], DNetDB [28], GTR, CNVD, Ensembl, GenCode, Novoseek, Swiss-Prot, LncRNADisease, Orphanet, and Catalogue Of Somatic Mutations In Cancer (COSMIC) [29]. Our gene datasets consist of 59,293 total genes (19,989 are protein-coding and 39,304 are non-protein-coding) and over 200,000 gene-disease combinations. We have integrated this high-volume and diverse database with GVViZ to support variable and complex gene-disease annotation, visualization, and expression analysis.

GVViZ is based on a product-line architecture, which means each module performs its task independently and its output is used as an input for the next module until the analysis outcome is achieved. It is a multi-platform software application programmed in JAVA, designed following the software engineering principles and the “Butterfly” paradigm [30]. GVViZ has been well tested and can be executed in Microsoft Windows, Linux, Unix, and MacOS operating systems. Along with user guidelines, further GVViZ database design and software development details are available in supplementary material 1.

## Results

**Fig. 4:**
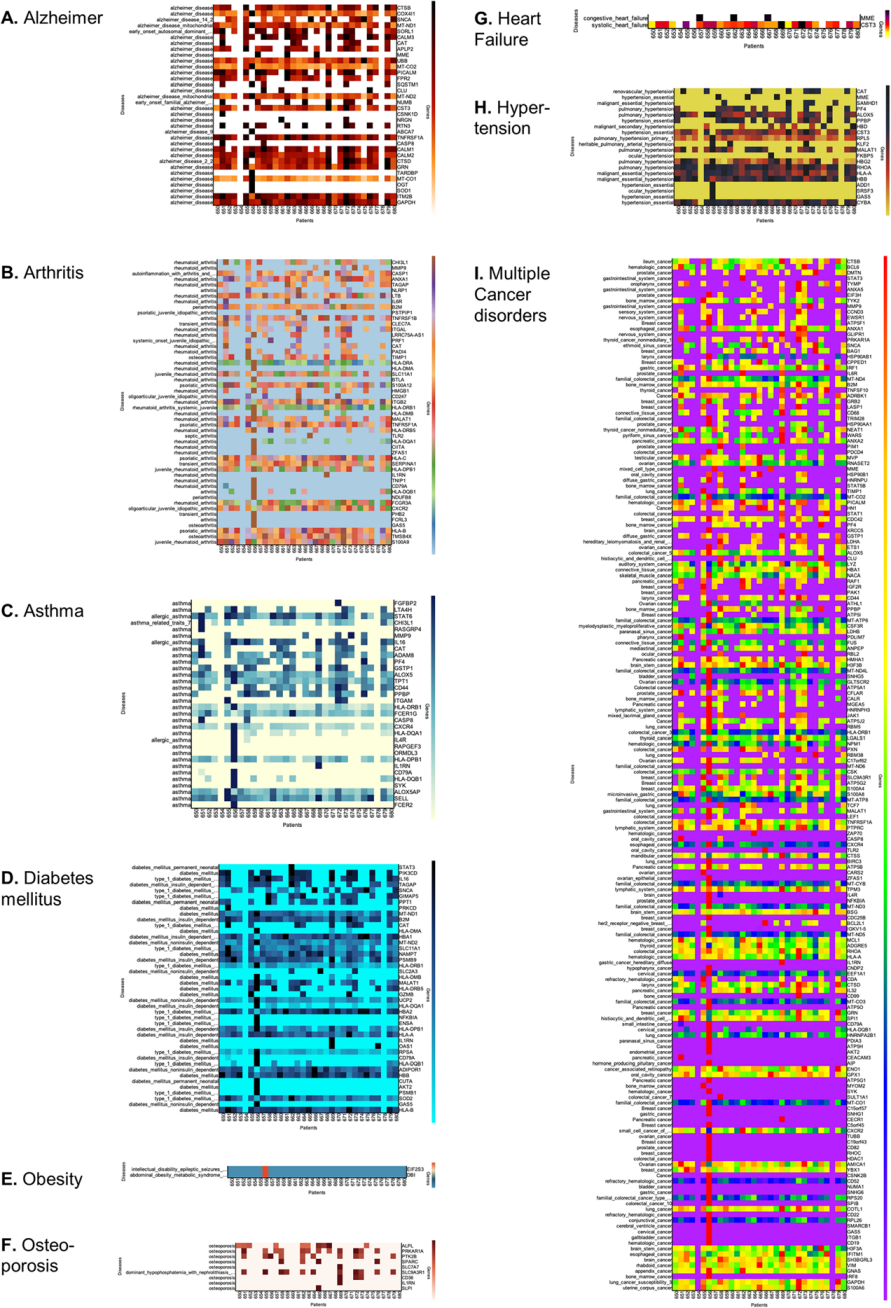
GVViZ gene-disease annotations, expression analyses, and heat map visualizations of different chronic diseases and conditions. Genes identified for **A** Alzheimer’s disease, **B** arthritis, **C** asthma, **D** diabetes mellitus, **E** obesity, **F** osteoporosis, **G** heart failure, **H** hypertension, and **I** multiple cancer disorders

The performance of GVViZ has been tested and validated in-house with multiple experimental analyses. Here, we report gene-disease annotation, expression mapping, and heat map visualization of different chronic diseases and conditions. We have integrated an annotated gene-disease database with a lab-generated dataset of randomly collected 31 RNA-seq samples. These patients were randomly selected for sequencing to explore variability in expression between individuals. We classified our analysis with an expression cutoff of 100 TPM for any gene in a single sample and mapped them to the physiological conditions: Alzheimer’s disease, arthritis, asthma, diabetes mellitus, obesity, osteoporosis, heart failure, hypertension, and multiple cancer disorders (Fig. 4). The annotation and expression analysis performed by GVViZ produced results linking expression genes to more than one chronic diseases, which included 34 genes linked to Alzheimer’s disease, 51 genes to arthritis, 32 genes to asthma, 43 genes to diabetes mellitus, 2 genes to obesity, 9 genes to osteoporosis, 2 genes to heart failure, and 20 genes to hypertension, and 184 genes were found to be associated to multiple cancer disorders (Fig. 4). GVViZ-produced results (high-resolution figures) are available in supplementary material 2.

We observed variability in the expression of Alzheimer’s-related genes *CTSB*, *COX4I1*, *MT-ND1*, *CALM3*, *UBB*, *MT-CO2*, *PICALM*, *FPR2*, *MT-ND2*, *CST3*, *TNFRSF1A*, *CALM1*, *CALM2*, *CTSD*, *GRN*, *MT-CO1*, *ITM2B*, and *GAPDH* between patients (Fig. 4A), having significant expression among most of the samples analyzed. Similarly, we found variable clusters of genes associated with immune-mediated diseases like arthritis and asthma. Arthritis genes included *ANXA1*, *LTB*, *B2M*, *TNFRSF1B*, *HLA-DRA*, *SLC11A1*, *S100A12*, *ITGB2*, *HLA-DRB1*, *TNFRSF1A*, *HLA-C*, *SERPINA1*, *HLA-DB1*, *FCGR3A*, *CXCR2*, *HLA-B*, *TMSB4X*, and *S100A9* (Fig. 4B) and with significant expression. Asthma-related genes *STAT6*, *IL16*, *ADAM8*, *ALOX5*, *TPT1*, *CD44*, *PPBP*, *HLA-DRB1*, *FCER1G*, *HLA-DQB1*, *ALOX5AP*, and *SELL* had substantial expression (Fig. 4C). Most widely occurring diseases like diabetes mellitus (*PIK3CD*, *IL16*, *MT-ND1*, *B2M*, *HBA1*, *MT-ND2*, *SLC11A1*, *NAMPT*, *PSMB9*, *HLA-DRB1*, *UCP2*, *HBA2*, *HLA-DPB1*, *HLA-A*, *ADIPOR1*, *HBB*, *SOD2*, *HLA-B*) (Fig. 4D) and hypertension (*ALOX5*, *CTS3*, *RPL5*, *HBG2*, *RHOA*, *HLA-A*, *HBB*, and *CYBA*) (Fig. 4H) showed variable degree of expression in some genes. We only noticed *CTS3* as a significantly regulated gene linked to heart failure (Fig. 4G) and did not find any highly expressed genes among obesity (Fig. 4E) and osteoporosis (Fig. 4F) disorders.

While analyzing multiple cancer disorders (Fig. 4I), we found highly variable expression among genes implicated in cancer: *EEF1A1*, *GNAS*, *NPM1*, *PIM1*, and *RHOA* are known oncogenes; *H3F3A*, *PTPRC*, *SMARCB1*, and *B2M* are possible oncogenes; and *CASP8*, *JAK1*, and *PRKAR1A* are known tumor suppressor genes [31]. While some genes found are known cancer census genes (*RAF1*, *BCL6*, *STAT3*, *CCND3*, *EWSR1*, *HSP90AB1*, *LASP1*, *HSP90AA1*, *STAT5B*, *PICALM*, *NACA*, *CSF3R*, *FUS*, *H3F3B*, *CALR*, *MALAT1*, *LEF1*, *CXCR4*, *BIRC3*, *TPM3*, *CD79A*, *HNRNPA2B1*, *AKT2*, *SYK*, and *NUMA1*) [32], evidence of aberrated expression of these genes could be a pre-clinical indication for further assessment. A further analysis of age, gender, and clinical history can give a clear idea of why some genes are expressed in nearly all the patients and some are not expressing in any patient. Also, we see many patients expressing most of these disease genes and a few not expressing any at all. Given their old age and no previous diagnosis, these patients should be studied to detect the signs of early-onset diseases.

## Discussion

The quest to understand what causes chronic, acute, and infectious diseases has been a central focus of humankind since the beginning of scientific discovery [33, 34]. Our evolving understanding of the complex nature of diseases has led us to realize that to effectively diagnose and treat patients with these conditions, it is essential to utilize a precision medicine approach [35, 36]. By identifying the novel risk factors and disease biomarkers, precision medicine translates scientific discovery into clinically actionable personal healthcare [37–46]. A major barrier to the implementation of precision medicine is the data analysis requirement. Precision medicine requires progressive healthcare IT environments that can efficiently and rapidly integrate data from disparate groups with non-aligned formats to provide decision-making information to healthcare providers without massive amounts of computing time [47]. Despite current progress, there is still no stand-alone platform available to efficiently integrate clinical, multi-omics, environmental, and epidemiological data acquisition [48]. Robust platforms are required in clinical settings to effectively manage, process, integrate, and analyze big data with variable structures [49, 50].

On-demand access and analysis of integrated and individual patient clinical and transcriptomics data can lead to the identification of diagnostic signatures for early dissemination of oncology and non-oncology disorders [51, 52]. It can support better aligning of known disease biomarkers with established treatments necessary for real-time personalized care. However, one of the existing challenges includes timely high-throughput genomics and transcriptomics data interpretation and visualization to support health practitioners in the provision of personalized care [53]. It requires integration and understanding of data with various types, structures, velocity, and magnitude [54]. Visualization of complex and high-volume data in health-related settings will support cognitive work and highly impact time-restricted decision-making [55]. Guidelines for the development of such applied and practical data visualization include but not limited to the implementation of an interactive and friendly user interface [30], efficient mapping of data elements to visual objects [55], use of easy to understand and self-explanatory data visualization techniques, exporting and sharing of produced results, flexible design available with open-source code, and most importantly based on a reproducible approach. The development of academic data visualization approaches will also contribute to improve the collaboration between computational and bench scientists and clinicians to practice precision medicine with impactful scientific discovery and accessible approach at the point of care [56].

In this manuscript, we presented GVViZ, an integrated computational platform to support population and personalized transcriptome analysis with a user-friendly, physician-oriented interface, and essential processes required for RNA-seq-driven gene expression modeling, analysis, integration, management, and visualization. GVViZ is particularly appropriate for demanding clinical settings to facilitate physician’s decision-making. As it offers integrating and using large amounts of transcriptomics generated, and gene-disease annotation data are collected to support the personalized care of individuals with several complex disorders. GVViZ has the potential to bring gene-disease data annotation and analytics to the bedside to facilitate genetic susceptibility for achieving truly personalized treatments for earlier, more effective disease intervention. We emphasize that automated graphical visualization should be an indispensable component of modern RNA-seq analysis, which is currently not the case [57–59]. However, researchers can use our interactive RNA-seq visualization tool to visualize the transcriptomics data making it a powerful tool to study the dynamics of gene expression and regulation. Integration of this tool into clinical settings can help generate a patient’s profile for precision medicine implementation. We used real RNA-seq data to show that our tool can help readily and robustly visualize patterns and problems that may give insight into a patient’s genomic profile, unravel genetic predisposition, and uncover genetic basis of multiple disorders.

The current release of the GVViZ does not support unsupervised gene expression and differential analysis. This is one of the very important aspects that we are looking forward to address in the future. Furthermore, we are planning to account for the potentially varying average transcript length across samples when performing differential gene expression analysis by scaling the TPM matrix (summing the estimated transcript TPMs within genes and multiplying with the total library size in millions). This will transform the underlying abundance measures to incorporate the information provided by the sequencing depth which may considerably improve the false discovery rate.

## Conclusion

Here, we introduced GVViZ, a new user-friendly application for RNA-seq-driven gene-disease data annotation, and expression analysis with a dynamic heat map visualization. With successful deployment in clinical settings, GVViZ will enable high-throughput correlations between patient diagnoses based on clinical and transcriptomics data. It will also assess genotype-phenotype associations among multiple complex diseases to find novel highly expressed genes. By mapping known and novel protein-coding and non-coding genes to their respective diseases, GVViZ can efficiently support the interpretation of genetic variants using the American College of Medical Genetics and Genomics (ACMG) guidelines and evaluation of variants in known genes.

## Availability and requirements

The software executable (JAR file) is open source and freely available. To execute GVViZ ver.1.0.0, the only requirement is the installation of Java Runtime Environment and MySQL. Once Java and MySQL have been installed, the following two tables need to be created in the MySQL server.

Operating system: Cross-platform (Microsoft Windows, MAC, Unix, Linux)

Programming languages: Java and MySQL

Requirements: The researcher is responsible for MySQL installation and  database schema.

License: Freely distributed for global users. Any restrictions to use by non-academics: Copyrights are to the authors.

Download link: GVViZ executable (JAR file) is freely available and can be downloaded through GitHub (https://github.com/drzeeshanahmed/GVViZ-Public).

GVViZ source code and all related material are already uploaded to GitHub and freely available to the community (https://github.com/drzeeshanahmed/GVViZ_SourceCode).

GVViZ online tutorial (video) is available through the following link: https://www.youtube.com/watch?v=x0RroYpk8Nw&ab_channel=Zeeshan.

## Supplementary Information

**Additional file 1: Supplementary material 1.** GVViZ: User guide, database modelling, source code and software configuration.

**Additional file 2: Supplementary material 2.** GVViZ produced results and high-resolution figures, and quality report by RNA-seq pipeline.

## Data Availability

The data that support the findings of this study are openly available in the following GitHub repository: https://github.com/drzeeshanahmed/GVViZ-Public. The data used in the current study are available from the corresponding author on reasonable request.

## Abbreviations

ACMG: American College of Medical Genetics and Genomics
COSMIC: Catalogue of Somatic Mutations in Cancer
DEGs: Differentially expressed genes
FAIR: Findable, accessible, interactive, and reusable
FPKM: Fragments per kilobase million
GUI: Graphical user interface
NGS: Next-generation sequencing
RPKM: Reads per kilobase of transcript per million mapped reads
RSEM: RNA by expectation maximization
RNA-seq: RNA sequencing
TPM: Transcripts per million
GVViZ: Visualizing genes with disease-causing variants
