# Supplementary material for: Advancing clinical genomics and precision medicine with GVViZ: FAIR bioinformatics platform for variable gene-disease annotation, visualization, and expression analysis
Source: Hum Genomics. 2021 Jun 26;15:37. doi: 10.1186/s40246-021-00336-1 (PMC8235866; doi:10.1186/s40246-021-00336-1)
Supplement: Supplementary file 1 — Additional file 1: Supplementary material 1. GVViZ: User guide, database modelling, source code and software configuration. [file 40246_2021_336_MOESM1_ESM.pdf]

Supplementary material 1:

GVViZ: User guide, database modelling, source code and software configuration.

Title

Advancing Clinical-Genomics and Precision Medicine with GVViZ: FAIR Bioinformatics Platform for Variable Gene-Disease Annotation, Visualization, and Expression Analysis

Authors

Zeeshan Ahmed[1, 2, *], Eduard Gibert Renart[1], Saman Zeeshan[3], XinQi Dong[1, 2]

Affiliations

1. Rutgers Institute for Health, Health Care Policy and Aging Research, Rutgers University, 112 Paterson Street, New Brunswick, NJ, USA.
2. Department of Medicine, Robert Wood Johnson Medical School, Rutgers Biomedical and Health Sciences, 125 Paterson Street, New Brunswick, NJ, USA.
3. Rutgers Cancer Institute of New Jersey, Rutgers University, 195 Little Albany St, New Brunswick, NJ, USA.

Corresponding author:

Zeeshan Ahmed (zahmed@ifh.rutgers.edu)

Table of Contents

GVViZ *3*
1. GVViZ: Main *3*
2. GVViZ GUI: Menu *6*
3. GVViZ GUI: Data Settings Panel *7*
4. GVViZ GUI: Data Visualization Panel *9*
5. GVViZ GUI: Heat Map Settings Panel *13*
GVViZ Database Design *17*
RNA-seq pipeline and annotated gene-disease data *18*
GVViZ Source Code – Class Design *19*
Availability and requirements *22*
Declarations *22*
Acknowledgements *22*
References *23*

GVViZ

GVViZ is a user-friendly desktop-bioinformatics application, developed by Ahmed Lab to support RNA-seq driven gene expressions, regulation, and disease annotation analysis with dynamic heat map visualization. It is based on set of simple instructions, following those a user without strong bioinformatics background and programming skills can perform gene expressions, regulation, and disease annotation analysis, and produce dynamic heat map visualization.

GVViZ is a multi-platform software package programmed in JAVA, designed following software engineering principles and “Butterfly” paradigm [1, 2]. It can execute on Microsoft Windows, Linux, Unix and macOS operating systems. Overall graphical user interface (GUI) of GVViZ consists of five main components:

1) Main
2) Menu
3) Data Settings Panel
4) Data Visualization Panel
5) Heat Map Settings Panel

1. GVViZ: Main

Main is the primary user interface of the GVViZ. As presented in **S. Figure 1**, and **S. Table 1**, it consists of a menu and three other panels:

1. Data Settings
2. Data Visualization
3. Heat Map Settings Panel

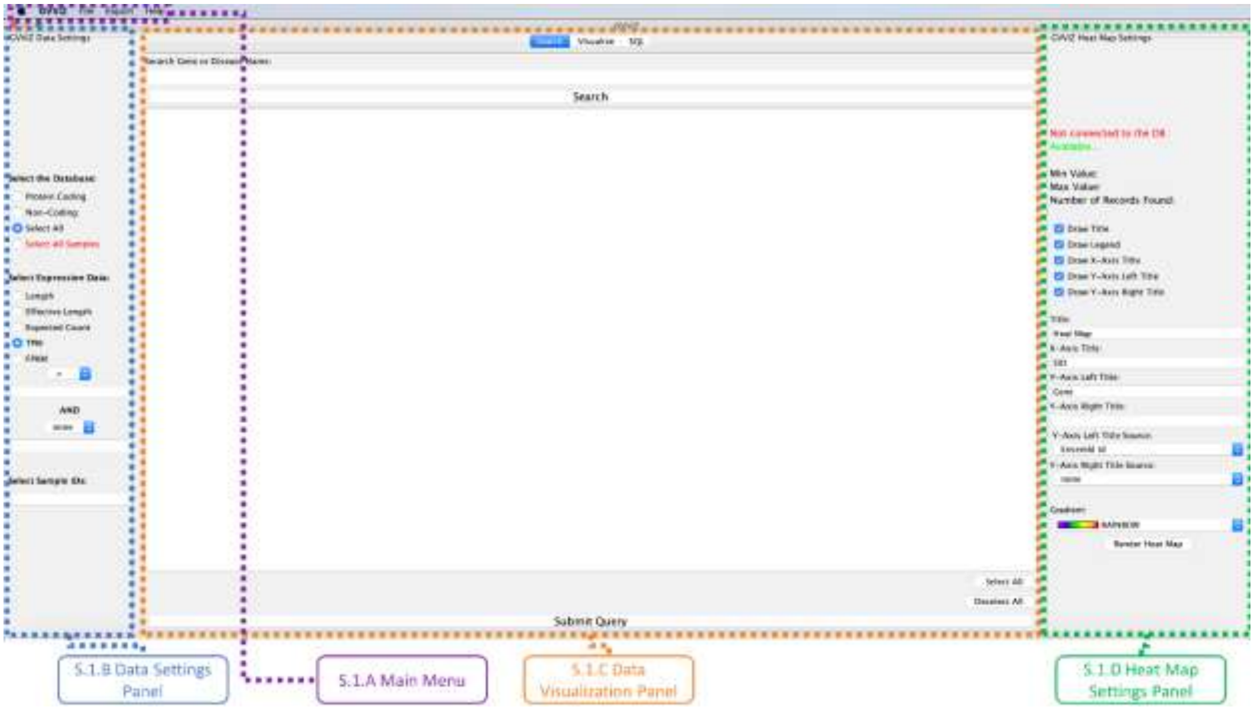

S. Figure 1. GVViZ: Main user interface.

No.	Feature	Description
S.1.A	Menu	Main top-down menu consists of three components:<br>1. File<br>• Navigates to Connect to the database panel.<br>• Provides option to Exit from GVViZ.<br>2. Export<br>• Provides options to export results:<br>▪ Text in CSV format file.<br>▪ Visualization in TIFF and PNG format files.<br>3. Help<br>• Provides author's Contact and GVViZ's About information
S.1.B	Data Settings Panel	The data settings panel offers three data selection options:<br>1. Select the database:<br>It allows user to choose between "Protein Coding", "Non-Coding", and all genes available in the integrated annotation database. It also allows user to select and analyze all available genes in the samples used for the analysis.<br>2. Select expression data:<br>It offers user to select expression value for the analysis. Current options include:<br>• Length<br>• Effective Length<br>• Expected Count<br>• TPM (Transcripts Per Kilobase Million)<br>• FPKM (Fragments Per Kilobase Million)<br>Additionally, it allows to set conditions to classify and filter results.<br>3. Select sample IDs.<br>It offers user to enter the IDs of the samples, user would analyze. It allows user to enter multiple commas separated samples (e.g., 1, 2, 3), as well

		as samples with rang (e.g., 1-10), and even both (e.g., 1,2,3, 5-15)
S.1.C	Data Visualization Panel	The data settings panel consists of three tabs:<br>1. Settings:<br>This panel allows user to search for genes and diseases to annotate, and genes and their computational values to collect from samples to perform visualization. Well explained in later sections.<br>2. Visualize:<br>This panel allows user to produce, render, and customize heatmaps.<br>3. SQL:<br>This panel allows user to manually run database queries and search for genes and diseases in database to annotate and curate data for visualization. Well explained in later sections.
S.1.D	Heat Map Settings Panel	The heat map settings panel offers customization of different values to support rendering, analysis, and visualization:<br>• Min Value<br>• Max Value<br>• Database connection<br>• Processing status<br>• Number of records found.<br>• Draw Title<br>• Draw Legend<br>• Draw X-Axis Title<br>• Draw Y-Axis Left Title<br>• Draw Y-Axis Right Title<br>• Title<br>• X-Axis Title<br>• Y-Axis Left Title<br>• Y-Axis Right Title<br>• Y-Axis Left Title Source<br>• Ensemble ID

		• Gene Name<br>• Disease Name<br>• Y-Axis Right Title Source<br>• None<br>• Ensemble ID<br>• Gene Name<br>• Disease Name<br>• Gradients<br>• Render Heat Map

S. Table 1. GVViZ: Features of Main user interface.

GVViZ basic workflow starts with the establishment of connection to the database by using the top menu, selecting connect to the database, provision of valid username, password, and address to the host database. Next, data needs to be searched and selected. Heatmap can be customized, as the user can set a new title, the number of y-axis and the gradient for the heat map. The last step is to visualize and export the rendered heat map.

2. GVViZ GUI: Menu

The main menu is available at the top of the GUI. It consists of three components (S. Figure 2 and S. Table 2).:

1. **File:**

   The file component allows user to connect to the local database and exit from the program. GVViZ relies on MySQL database management system (server), where gene-disease annotation and expression data are stored to support data analysis and heat map visualization. User needs to connect to the database by clicking on the connect to database button. With that a popup window will appear, where the user is required to input the database connection related information (hostname, username, password, and port).

2. **Export:**

   The export option allows user to save the heat maps generated in two different image formats (PNG and TIFF). In addition, the user has the option to save the matrix produced by GVViZ into a CSV file.

3. **Help:**

   Help is the last component in the main menu. It consists of two options: About and Contact Us. The about displays information about the current version of GVViZ and Contact Us displays information to contact the authors.

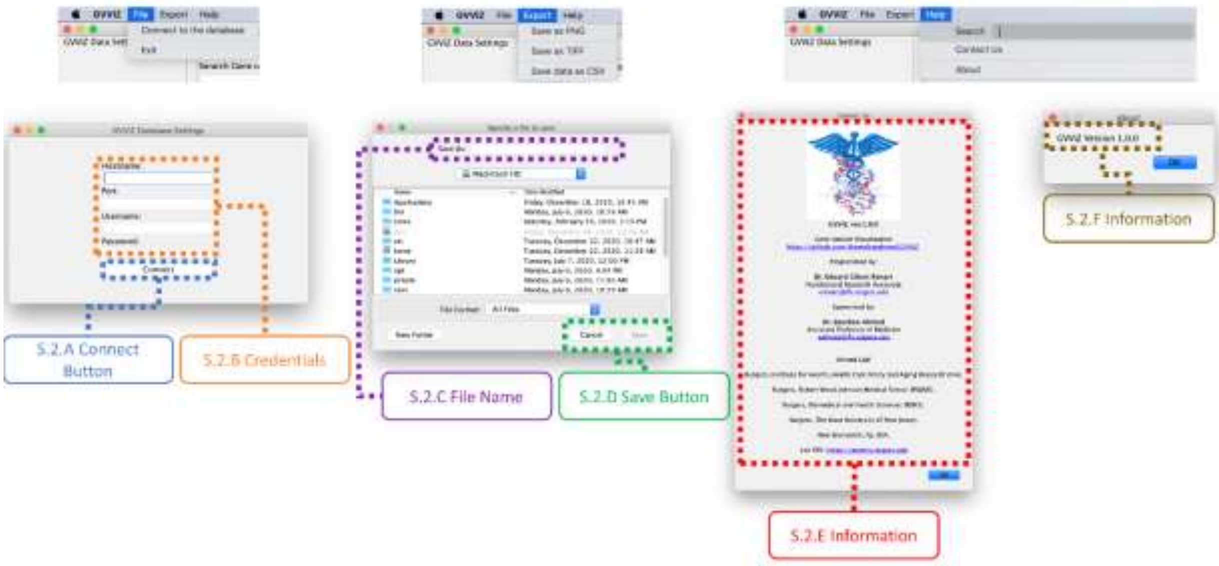

S. Figure 2. GVViZ main menu.

No.	Feature	Description
S.2.A	File	• Connect to database: To connect to the local database.<br>• Exit: To terminate the program.
S.2.B	Export	• Export to PNG: Save the heat map as PNG.<br>• Export to TIFF: Save the heat map as TIFF.<br>• Save data as CSV: Save the heat map as CSV.
S.2.C	Help	• Contact us: Popup that displays information for contacting the authors of the GVViZ.<br>• About: Popup that displays information about the current version of the GVViZ.

S. Table 2. GVViZ main menu.

3. GVViZ GUI: Data Settings Panel

The data settings panel is in the WEST side of the screen and it is divided into three sections (S. Figure 3 and S. Table 3):

1. **Type**:

   In this section the user needs to select the type of data to consume. The user has four options available to choose from: 1) select protein coding genes, 2) select non-protein coding genes, 3) select all (protein and non-protein coding genes), and 4) select all the samples stored in the database.

2. **Expression selection:**

There are five different gene expression metrics to choose from: 1) length, 2) effective length, 3) expected count, 4) TPM, and 5) FPKM. The user can impose up to two constraints that can be applied to select the gene expression data. The following are all the operators that can be used to impose constraints in the data: “>”, “<”, “=”, “<=”, “>=”, “! =”. Lastly, the user is required to input a number in the text box to complete the constraint e.g., to plot heat map that contain samples with TPM greater than 50: user needs to first select TPM, then in the drop-down menu select the operator “>” and input 50 in the text box. This will only select samples stored in the database in which their TPM values are higher than 50.

3. **Sample IDs:**

After selecting the expression data, the next step is to select the sample ids that need to be visualized in the heat map. To do that the user needs to input all the sample ids separated by a “,” or by a “-” to plot a range in the text box provided. The combination of both “,” and “-” are also considered valid inputs e.g., to plot a heatmap with samples ranged from 10 to 100, the expression needs to look like: 10-100.

**No.**	**Feature**	**Description**
S.3.A	Type	• Protein Coding genes.<br>• Non-Coding genes<br>• All genes from database<br>• All genes from all samples
S.3.B	Expression data	• Length<br>• Effective Length<br>• Expected Count<br>• TPM: Transcripts Per Kilobase Million<br>• FPKM: Fragments Per Kilobase Million<br>• “>, <, =, <=, >=, !=:” Possible data constraints.<br>• Text box to input the constraint.<br>• “None, >, <, =, <=, >=, !=:” Additional data constraints.<br>• Text box to input the second constraint.
S.3.C	Sample ids	• Text box to input the sample ids to select.

S. Table 3. GVViZ data settings panel.

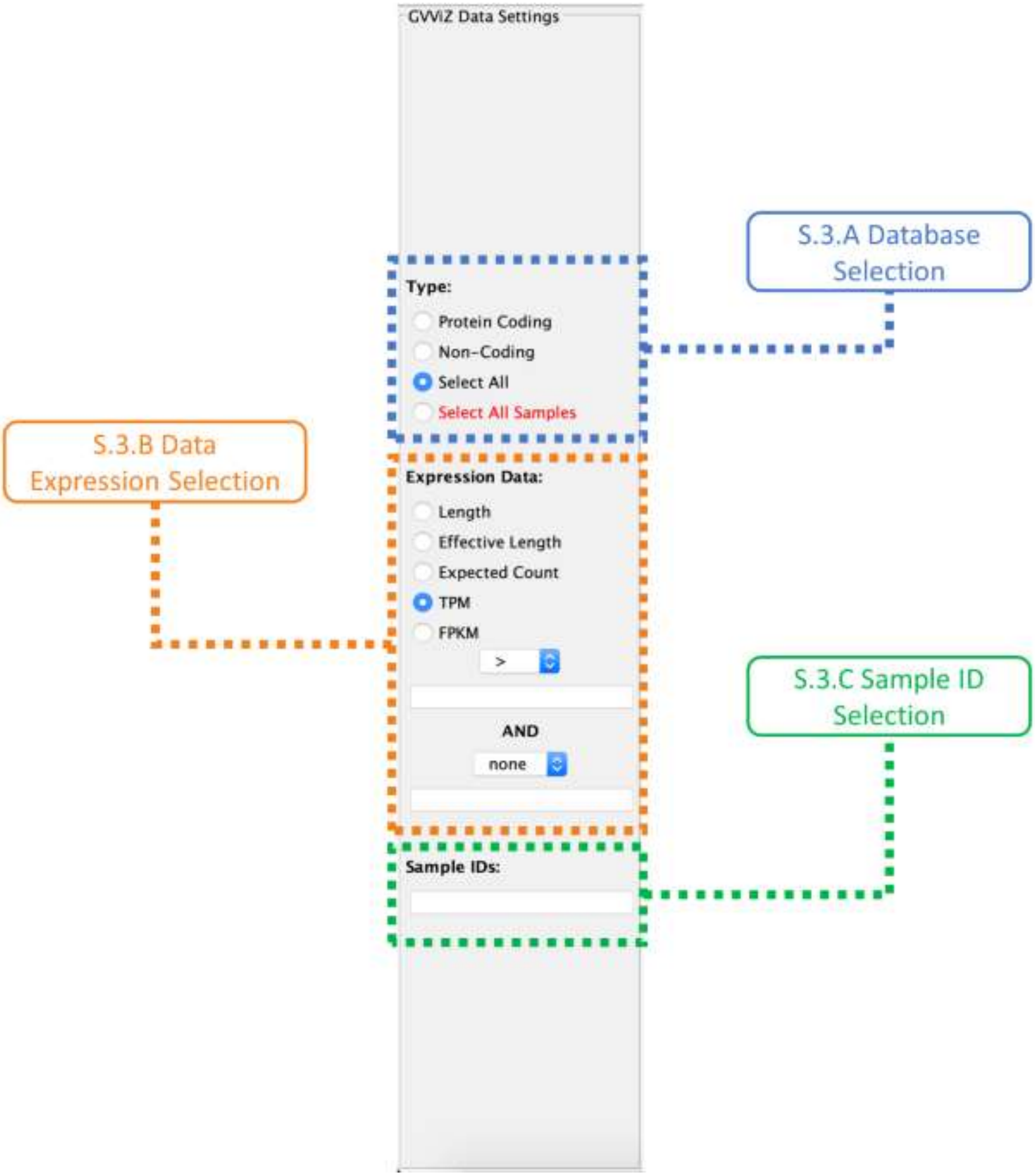

S. Figure 3. GVViZ data settings panel.

4. GVViZ GUI: Data Visualization Panel

Once the user has selected the data, the next step is to select genes to plot heat map using data visualization panel. The data visualization panel is divided into three tabs/modules:

GVViZ © Ahmed Lab, Rutgers University, 2021.

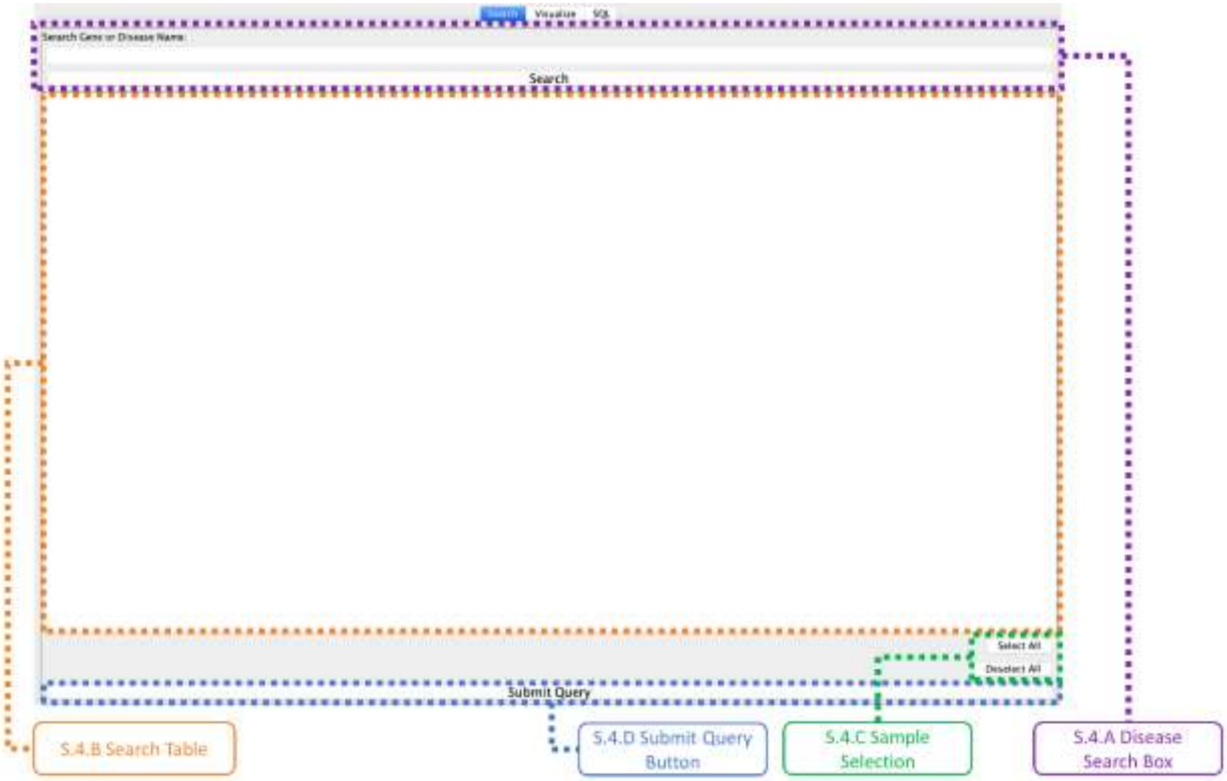

S. Figure 4. GVViZ search screen located in the center of the screen.

No.	Feature	Description
S.4.A	Disease Search Box	• Text box for typing disease keywords.<br>• Search button to submit query.
S.4.B	Search Table	• Results from the search box will appear here.
S.4.C	Sample Selection	• Select all samples.<br>• Deselect all samples.
S.4.D	Submit Query Button	• Submits the overall query to render the heat map.

S. Table 4. GVViZ search screen features.

1. **Search:**

The search tab allows user to look for genes that are associated to diseases. The user needs to input disease keyword (full or partial) in the text bar and click the search button. A collection of genes that are related to that disease will appear in. This information will be based on the backend connected gene-disease annotation database. Having the

list of genes at successful execution of automatically generated SQL query, user can select all genes and customize selection to submit query to start rendering of the heat map (S. Figure. 5 and S. Table 4).

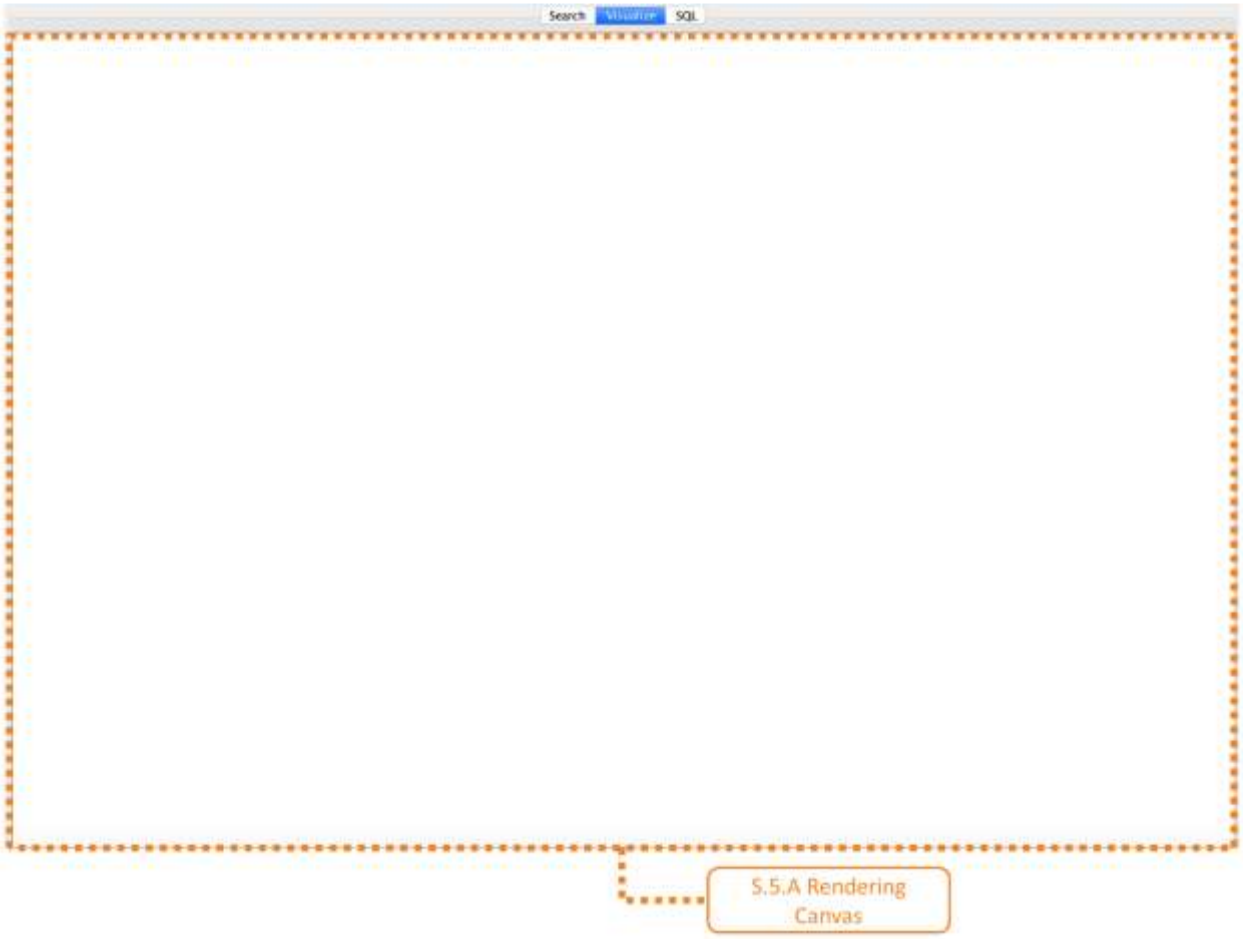

S. Figure 5. GVViZ visualization screen located in the center of the screen.

No.	Feature	Description
S.5.A	Rendering Canvas	• The heat map will be rendered here.

S. Table 5. GVViZ visualization screen features

2. **Visualization:**

This module is to displays the heat map. Every time, when a new/change is made to the heat map, the render heat map button needs to be clicked to draw/refresh heat map (S. Figure. 5 and S. Table 5).

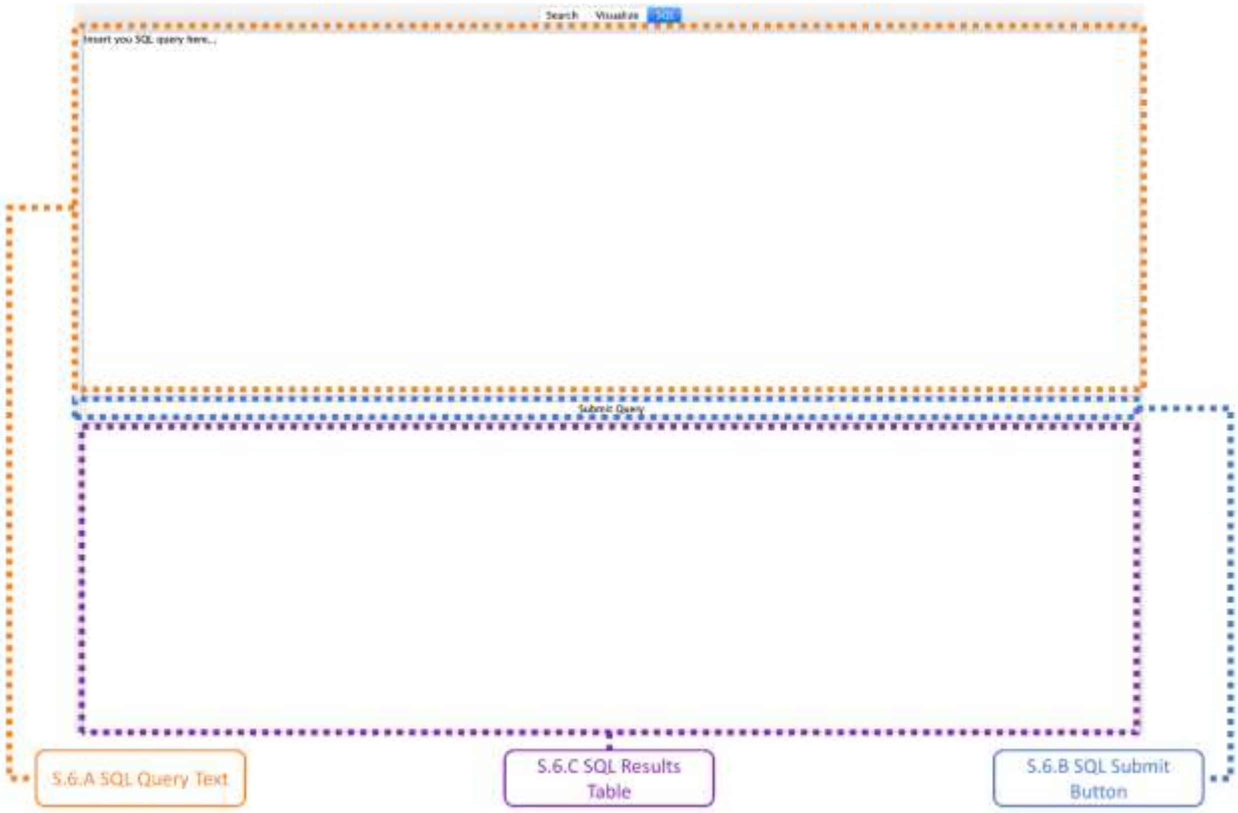

S. Figure 6. GVViZ SQL screen located in the center of the screen.

No.	Feature	Description
S.6.A	SQL Query Box	• SQL query to send to the database.
S.6.B	SQL Submit Button	• Submit the query inputted in the text box.
S.6.C	SQL Results Table	• Results of the SQL query will appear in this table.

S. Table 6. GVViZ SQL screen features

3. SQL:

The last tab is the SQL that offer features to perform SQL queries directly with the database. The text box located in the top of the interface allows user to input the SQL query to be executed. In the middle of the interface, there is the button to submit the query, and in the bottom of the interface, there is a table where the output of the SQL query will be displayed (S. Figure. 6 and S. Table 6).

5. GVViZ GUI: Heat Map Settings Panel

The last panel is the heat map customization that is in the EAST side of the screen and allows user to render and customize the looks of the heat map (S. Figure.7 and S. Table 7). This panel is divided into five sections:

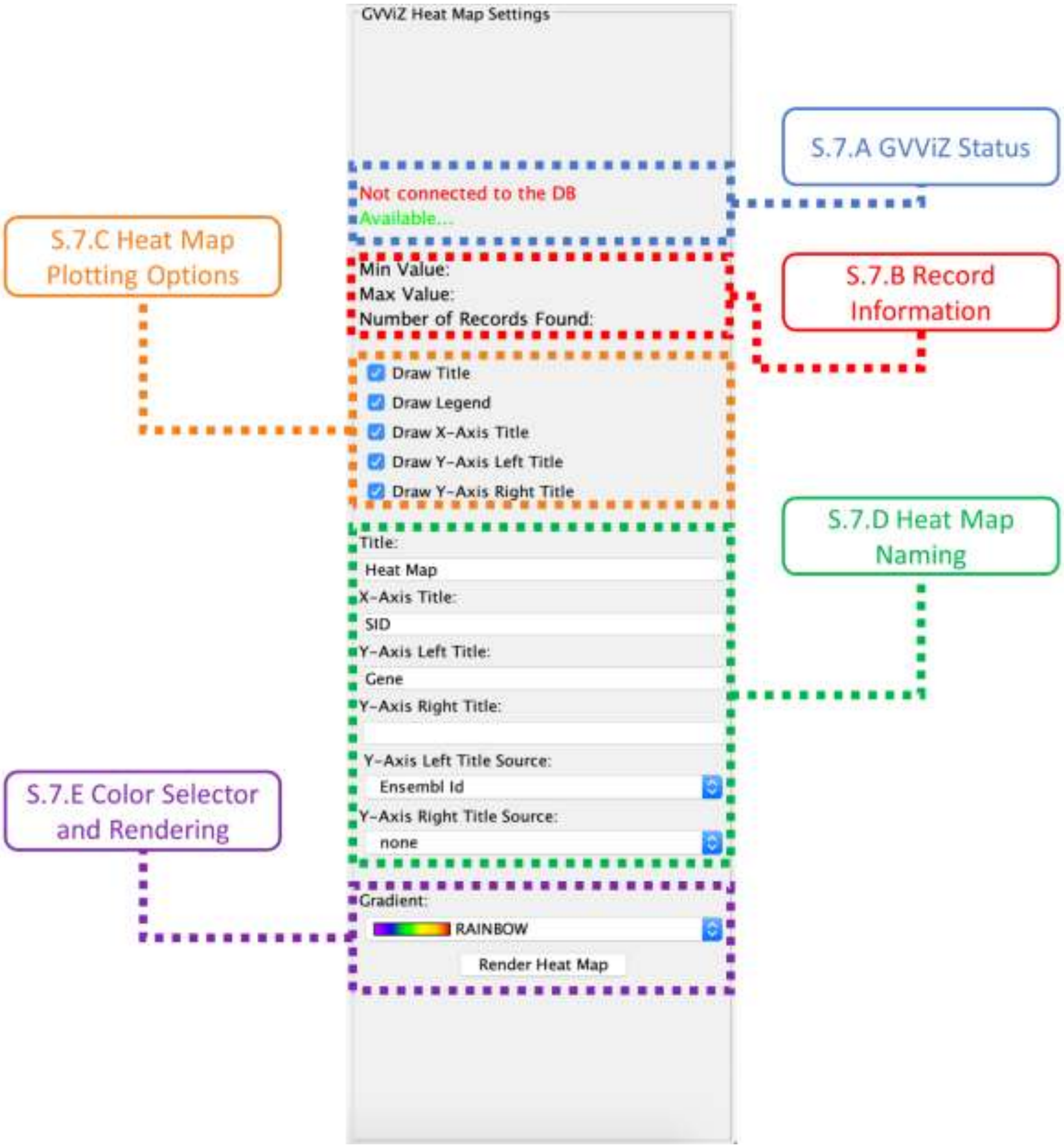

S. Figure 7. GVViZ heat map screen located in the EAST of the screen.

No.	Feature	Description
S.7.A	GVViZ Status	• Database status connection: it displays if GVViZ is currently connected to the local database.<br>• Processing status: displays if GVViZ is performing computations on the background or if its idle.
S.7.B	Record Information	• Min Value: displays the smallest value that is currently being rendered in the heat map.<br>• Max Value: displays the biggest value that is currently being rendered in the heat map.<br>• Number of records found: displays the total number of records that where returned by the keyword that was inputted in the disease search box.
S.7.C	Heat Maps Plotting Options	• Draw Title: checkbox to display or not display the heat map title.<br>• Draw Legend: checkbox to render the legend for the heat map.<br>• Draw X-Axis Title: checkbox to display or not display the x-axis title of the heat map.<br>• Draw Y-Axis Left Title: checkbox for rendering or not rendering the left y-axis of the heat map.<br>• Draw Y-Axis Right Title: checkbox for rendering or not rendering the right y-axis of the heat map.
S.7.D	Heat Map Naming.	• Title: text box for setting the title name of the heat map.<br>• X-Axis Title: text box for setting the x-axis title name.<br>• Y-Axis Left Title: text box for setting the left y-axis title name.<br>• Y-Axis Right Title: text box for setting the right y-axis title name.<br>• Y-Axis Left Title Source<br>• Ensemble ID

		• Gene Name<br>• Disease Name<br>• Y-Axis Right Title Source<br>• None: do not display anything<br>• Ensemble ID: display the ensemble id<br>• Gene Name: display the gene name<br>• Disease Name: display the disease name
S.7.E	Color Selector and Rendering	• Gradients: a collection of color gradients that can be used to render different types of heat maps.<br>• Render Heat Map, it needs to be clicked to render the heat map.

S. Table 7. GVViZ heat map screen features

1. **Status:**

   The first section of the panel are two labels that communicate the status of the connection with the local database and a status of the processing.

2. **Record information:**

   The second section consists of two informative labels that displays the minimum and the maximum values of the plotted heat map.

3. **Heat map plotting options:**

   The third section is a collection of five check boxes that allows the user to decide if to render the tile, the legend, the x-axis title or two render a single or a double y-axis. Selecting or deselecting one of this option will be reflect on the visualization tab after clicking the render heat map button, which is located on the bottom of this panel.

4. **Heat map naming:**

   The fourth section allows user to set a title name, x-axis title, y-axis titles and select the names to be displayed for both y-axis. Note that if none is selected for the y-axis right, it will only plot a single y-axis.

5. **Color selector and rendering:**

   The last section consists of two components are the color scheme selector for the heat map, where the user can select from twenty-eight different color combinations, and the render heat map button, which needs to be clicked every time a new change has been made to the heat map. The following table lists all the gradient combinations available, along with the high and the low color (S. Table 8).

**Gradient Name**	**High Color**	**Low Color**
RAINBOW	Purple	Red
BLUE_TO_RED	Blue	Red
GREEN_YELLOW_ORANGE_RED	Green	Red
HEAT	Light Brown	Black
HOT	Yellow	Black
MAROON_TO_GOLD	Maroon	Gold
WHITE_TO_BLACK	White	Black
GREEN_TO_RED	Green	Red
YOR	Yellow	Red
WHITE_TO_BLUE	White	Blue
PLANET_HEART	Blue	Black
HEATED_METAL	Yellow	Black
DEEP_SEA	Light Blue	Dark Blue
BU_GN_YI	Blue	Yellow
RED	White	Red
BLUE	White	Blue
DIVERGING	Blue	Red
DIVERGING2	Pink	Light Blue
PINK_GREEN	Pink	Green
YELLOW_TO_PURPLE	Yellow	Purple
ORANGE_PINK_GREY	Orange	Gray
BLUE_YELLOW_RED	Blue	Red
BLUE_YELLOW_RED	Blue	Red
BLUE_BROWN_RED	Blue	Red

GREEN_TO_ORANGE	Green	Orange
PAIRD	Blue	Purple
ACCENT	Green	Brown
ROCKET	Light Brown	Black

S. Table 8. GVViZ gradient color combinations.

GVViZ Database Design

GVViZ simplifies the process of visualization and exploration of results by using a SQL database where the results of the RNA-seq pipeline are automatically parsed and upload. Our gene expression database model consists of two relations/tables (S. Figure 8):

1. **Gene Expression Data:**

   The Gene Expression Data table contains all the data that has been populated by the RNA-seq data pipeline and uploaded to this table. This table is consisting of seven columns (S. Table 9, and S. Figure 8): gene id, length, effective length, expected count, TPM, FPKM and SID (sample id).

2. **Gene Disease Annotation:**

   The Gene Disease Annotation table is used for gene disease annotation. The table contains four columns: gene id, ensemble id, category, and the disease (S. Table 10 and S. Figure 8).

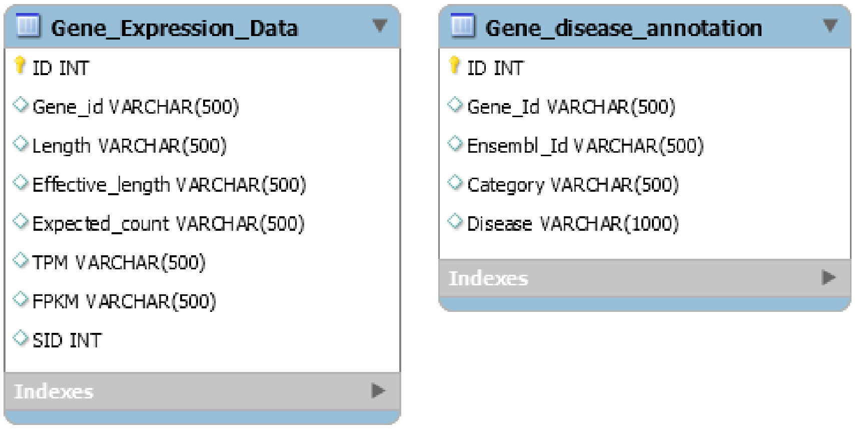

S. Figure 8. GVViZ entity relationship diagram (ERD).

Number	Name	Description
1	Gene_Id,	Ensembl Stable ID (String)
2	Length	Gene Length (String)
3	Effective_Length	Effective Length (String)
4	Expected_Count	Expected Count (String)
5	TPM	Transcripts Per Kilobase Million (String)
6	FPKM	Fragments Per Kilobase Million (String)
7	SID	Sample Id (Integer)

S. Table 9. GVViZ gene expression database schema.

Number	Name	Description
1	Gene_Id,	ID to identify the gene (String)
2	Ensembl_Id	Ensemble Stable ID (String)
3	Category	Either protein coding or non-protein coding (String)
4	Disease	Disease Name (String)

S. Table 10. GVViZ gene disease annotation database schema.

RNA-seq pipeline and annotated gene-disease data

A typical workflow for RNA-seq (S. Figure 9) analysis using GVViZ is as follows. First the RNA-seq pipeline is deployed, in which the quality control of the raw reads is conducted using FastQC [3]. Then the reads are trimmed using Trimmomatic [4], and the data sequences are sorted using SAMtools [5]. MarkDuplicates is then used for removing duplicates [16], and CollectInsertSizeMetrics are used to compute size distribution and read orientation of paired-end libraries. Then the paired end raw reads are aligned to the human reference genome (hg38) using HISAT [7] with Bowtie2 [8] software.

RNA by Expectation Maximization (RSEM) [9] is then applied for quantification and identification of differentially expressed genes by aligning reads to reference de novo transcriptome assemblies, based on TPM (Transcript Per Million mapped reads). Lastly the results of the RNA-seq pipeline are parsed and automatically loaded into the GVViZ gene expression database, where the results will be queried and visualized using the GUI of GVViZ.

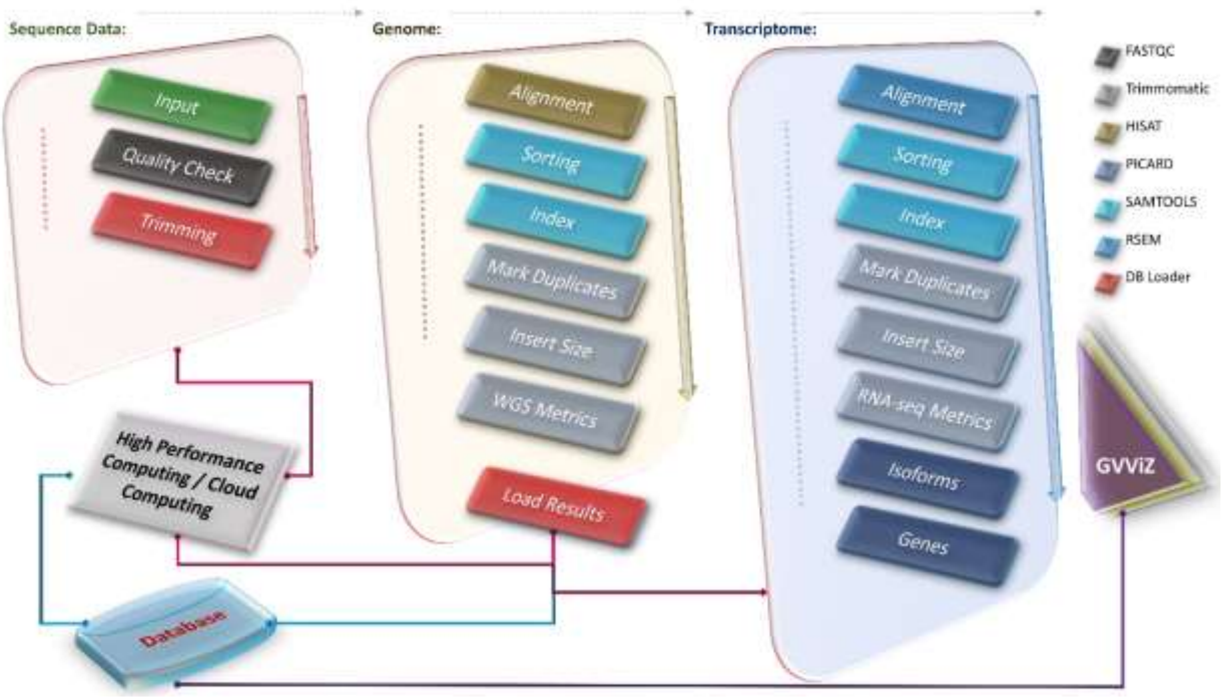

S. Figure 9. RNA-seq pipeline.

The gene disease annotation table can be populated with annotated gene-disease data, which can be collected from different clinical and genomics databases [10]. These databases can include but not limited to the PAS, ClinVar, GeneCards, MalaCard, DISEASES, HGMD, Disease Ontology, DiseaseEnhancer, DisGeNET, eDGAR, GTR, OMIM, miR2Disease, DNetDB, The Cancer Genome Atlas, International Cancer Genome Consortium, OMIM, GTR, CNVD, Ensembl, GenCode, Novoseek, Swiss-Prot, LncRNADisease, Orphanet, WHO, FDA, Catalogue of Somatic Mutations in Cancer (COSMIC) and Genome-wide Association Studies (GWAS) [10, 11, 12].

GVViZ Source Code – Class Design

Source code of GVViZ is based on total 15 classes: (S. Figure 8, and S. Table 11):

1. GVViZ
2. Gradient
3. ContactView
4. ExecuteQuery
5. SQLController
6. Heat Map
7. ExecuteQuery2
8. MenuController
9. SQLView
10. BeginerView2
11. Model
12. BeginerView
13. MenuView
14. DBViewFrame
15. MyTableModel

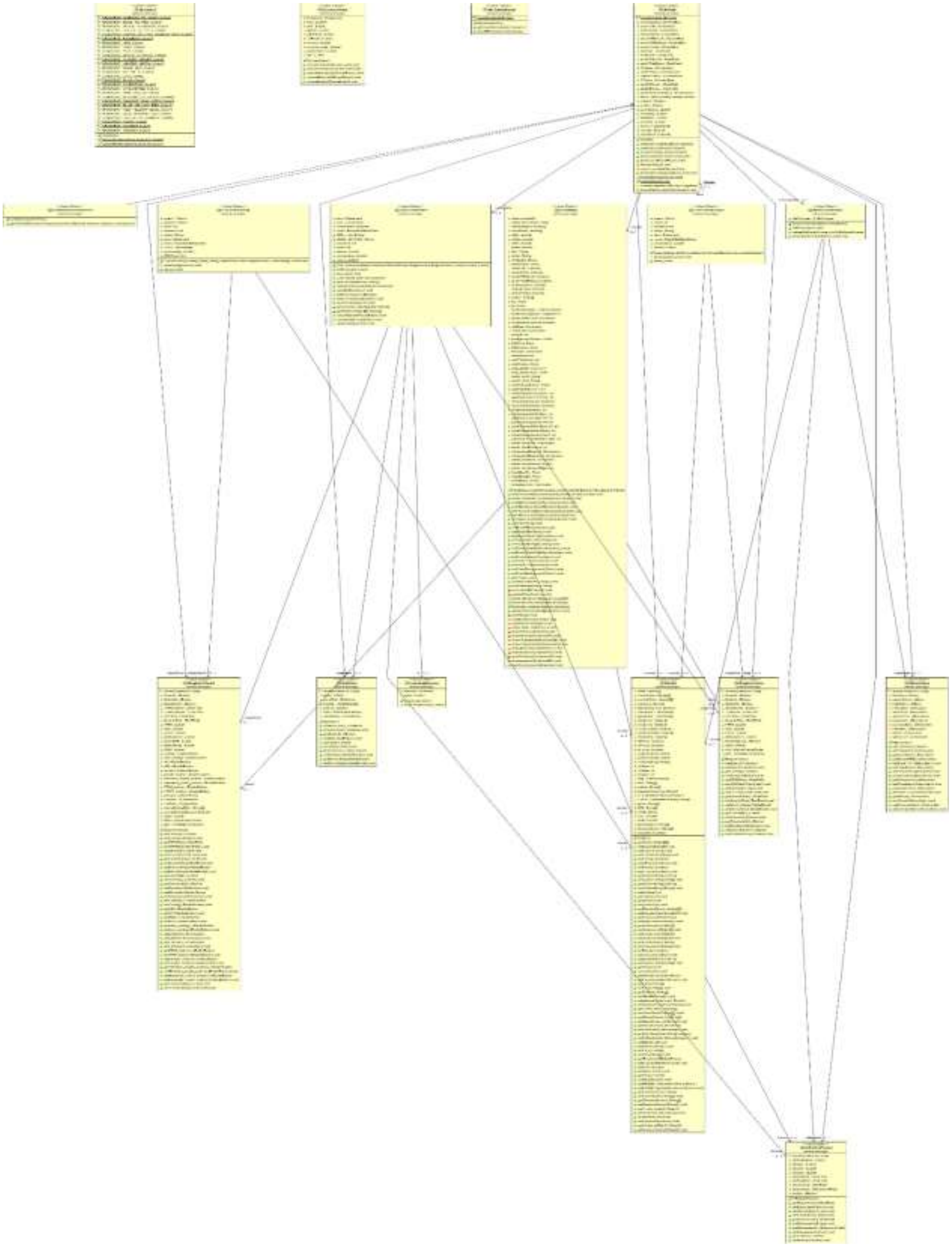

S. Figure 9. GVViZ class diagrams.

Number	Class	Description	Relationship with other classes
1	GVViZ	This is the main class	4-15
2	Gradient	Class that defines all the gradient colors.	N/A
3	ContactView	Class that defines the placement and objects part of the contact page.	N/A
4	ExecuteQuery	Class that contains the logic for executing the SQL queries for the SQL tap	11,12
5	SQLController	Class that manages the SQL connections to the database.	9,10,11,12,14
6	Heat Map	Class that is responsible for rendering and offering remaining customized features to produce heat maps.	10,12
7	ExecuteQuery2	Class that contains the logic for executing the complex SQL queries for the visualize tap.	11,12
8	MenuController	Class that implements the functionality of the top main menu.	11,12,13
9	SQLView	Class that defines the view of SQL tap.	N/A
10	BeginerView2	Class that defines the placements, and objects of the GVViZ data settings view.	N/A
11	Model	Class that temporarily stores all the data used in the program.	14
12	BeginerView	Class that defines the placement, and objects of the heat map settings.	N/A
13	MenuView	Class that defines the placement, and objects of the main menu.	N/A
14	DBViewFrame	Class that stores all the credentials for the database.	N/A
16	MyTableModel	Class that defines display of SQL table.	N/A

S. Table 11 GVViZ Class descriptions and relationships. N/A = Not Applicable.

Availability and requirements

The software executable (JAR file) is open source and freely available and to execute GVViZ ver.1.0.0 the only requirements is the installation of Java Runtime Environment and MySQL. Once Java and MySQL have been installed the following two tables need to be created in the MySQL server.

Operating system: Cross platform (Microsoft Windows, MAC, Unix)

Programming languages: Java and MySQL

Requirements: The researcher is responsible for having installed MySQL and having the provided database schema into the database.

License: Freely distributed for global users. Any restrictions to use by non-academics: Copyrights are to the authors.

Download link: GVViZ executable (JAR file) is freely available and can be downloaded through GitHub <https://github.com/drzeeshanahmed/GVViZ-Public>.

GVViZ source code and all related material is already uploaded to GitHub and will be made open / freely available to community, when paper will be published online. <https://github.com/drzeeshanahmed/GVViZ_SourceCode>

GVViZ online tutorial (video) Is available through following link: <https://www.youtube.com/watch?v=x0RroYpk8Nw&ab_channel=Zeeshan>.

Declarations

Ethical Approval and Consent to participate: Not applicable.

Consent for publication: Not applicable

Availability of data and material: The data that support the findings of this study are openly available in the following GitHub repository: <https://github.com/drzeeshanahmed/GVViZ-Public>

Competing interests: The Authors declare no Competing Financial or Non-Financial Interests.

Funding: This work was supported by the Institute for Health, Health Care Policy and Aging Research, and Robert Wood Johnson Medical School, at Rutgers, The State University of New Jersey.

Acknowledgements

We appreciate great support by the Institute for Health, Health Care Policy and Aging Research (IFH), and Rutgers Robert Wood Johnson Medical School, Rutgers Biomedical and Health Sciences at the Rutgers, The State University of New Jersey.

We thank members and collaborators of Ahmed Lab <https://promis.rutgers.edu/> at the Rutgers IFH for their active participation and contribution to this study.

This study was completed in part by research services and/or survey/data resources provided by the Institute for Health Survey / Data Core at Rutgers University, available at: <http://www.ifhcore.rutgers.edu>

The authors acknowledge the Office of Advanced Research Computing (OARC) at Rutgers, The State University of New Jersey for providing access to the Amarel cluster and

associated research computing resources that have contributed to the results reported here. URL: <https://it.rutgers.edu/oarc>

References

1. Ahmed, Z., Zeeshan, S., & Dandekar, T. (2014). Developing sustainable software solutions for bioinformatics by the " Butterfly" paradigm. F1000Research, 3, 71.
2. Ahmed, Z., Zeeshan, S. (2014). Cultivating Software Solutions Development in the Scientific Academia. Recent Patents on Computer Science. 7, 54-66.
3. Trivedi, U. H., et al. (2014). Quality control of next-generation sequencing data without a reference. Frontiers in genetics, 5, 111.
4. Bolger, A. M., Lohse, M., & Usadel, B. (2014). Trimmomatic: a flexible trimmer for Illumina sequence data. Bioinformatics (Oxford, England), 30(15), 2114–2120. https://doi.org/10.1093/bioinformatics/btu170
5. Li, H., et al. (2009). The Sequence Alignment/Map format and SAMtools. Bioinformatics (Oxford, England), 25(16), 2078–2079.
6. Ebbert, M. T., et al. (2016). Evaluating the necessity of PCR duplicate removal from next-generation sequencing data and a comparison of approaches. BMC bioinformatics, 17 Suppl 7(Suppl 7), 239. https://doi.org/10.1186/s12859-016-1097-3
7. Kim, D., Langmead, B., & Salzberg, S. L. (2015). HISAT: a fast spliced aligner with low memory requirements. Nature methods, 12(4), 357–360.
8. Langmead, B., & Salzberg, S. L. (2012). Fast gapped-read alignment with Bowtie 2. Nature methods, 9(4), 357–359.
9. Li, B., & Dewey, C. N. (2011). RSEM: accurate transcript quantification from RNA-Seq data with or without a reference genome. BMC bioinformatics, 12, 323. https://doi.org/10.1186/1471-2105-12-323
10. Zeeshan, S., Xiong, R., Liang, B. T., & Ahmed, Z. (2020). 100 Years of evolving gene-disease complexities and scientific debutants. Briefings in bioinformatics, 21(3), 885–905.
11. Ahmed, Z., Zeeshan, S., Mendhe, D., & Dong, X. (2020). Human gene and disease associations for clinical-genomics and precision medicine research. Clinical and translational medicine, 10(1), 297–318.
12. Ahmed, Z., Zeeshan, S., Xiong, R., & Liang, B. T. (2019). Debutant iOS app and gene-disease complexities in clinical genomics and precision medicine. Clinical and translational medicine, 8(1), 26.
