# Supplementary material for: Advancing clinical genomics and precision medicine with GVViZ: FAIR bioinformatics platform for variable gene-disease annotation, visualization, and expression analysis
Source: Hum Genomics. 2021 Jun 26;15:37. doi: 10.1186/s40246-021-00336-1 (PMC8235866; doi:10.1186/s40246-021-00336-1)
Supplement: Supplementary file 2 — Additional file 2: Supplementary material 2. GVViZ produced results and high-resolution figures, and quality report by RNA-seq pipeline. [file 40246_2021_336_MOESM2_ESM.pdf]

Supplementary material 2:

GVViZ produced results and high-resolution figures, and quality report by RNA-seq pipeline.

Title

Advancing Clinical-Genomics and Precision Medicine with GVViZ: FAIR Bioinformatics Platform for Variable Gene-Disease Annotation, Visualization, and Expression Analysis

Authors

Zeeshan Ahmed[1, 2, *], Eduard Gibert Renart[1], Saman Zeeshan[3], XinQi Dong[1, 2]

Affiliations

1. Rutgers Institute for Health, Health Care Policy and Aging Research, Rutgers University, 112 Paterson Street, New Brunswick, NJ, USA.

2. Department of Medicine, Robert Wood Johnson Medical School, Rutgers Biomedical and Health Sciences, 125 Paterson Street, New Brunswick, NJ, USA.

3. Rutgers Cancer Institute of New Jersey, Rutgers University, 195 Little Albany St, New Brunswick, NJ, USA.

Corresponding author:

Zeeshan Ahmed (zahmed@ifh.rutgers.edu)

GVViZ © Ahmed Lab, Rutgers University, 2021.

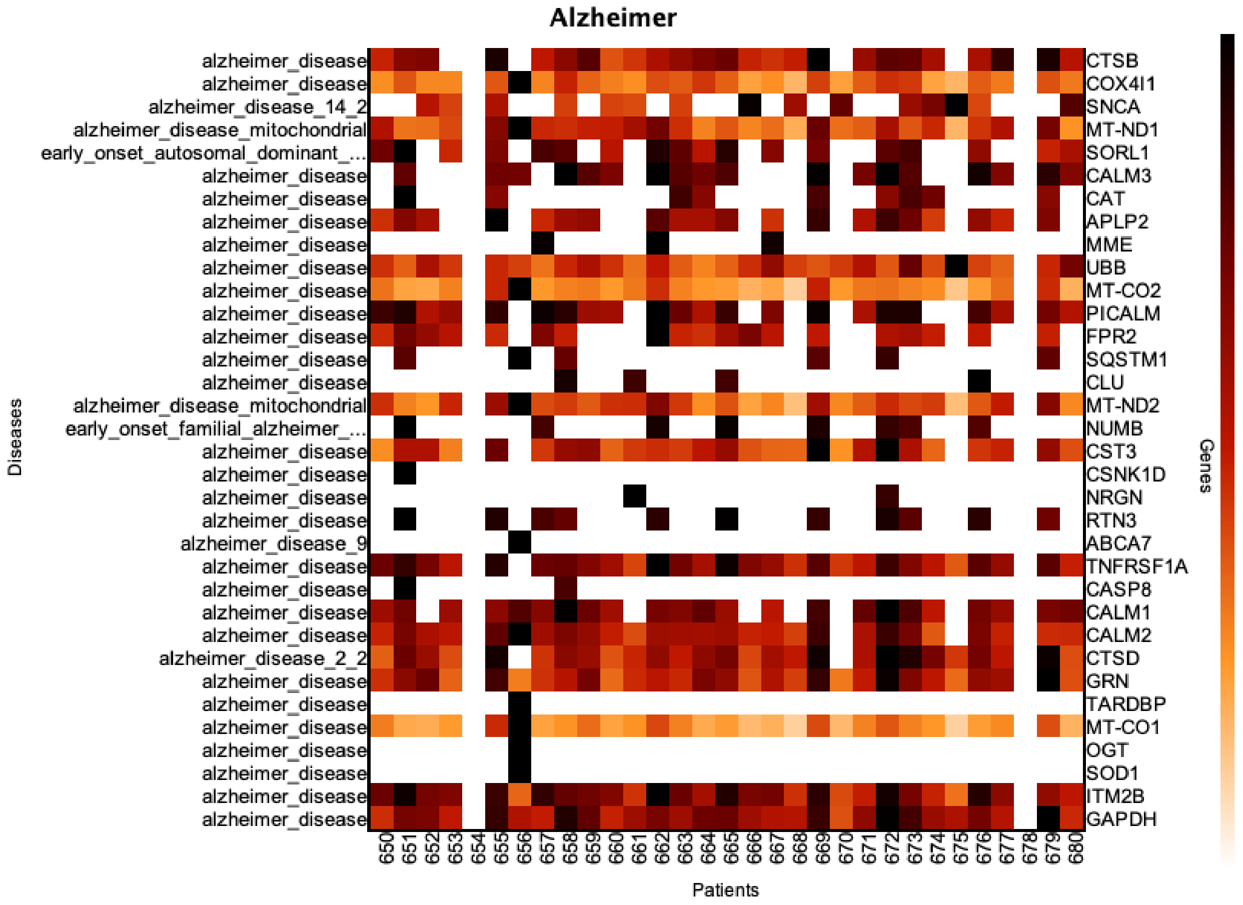

S. Figure 1. Alzheimer

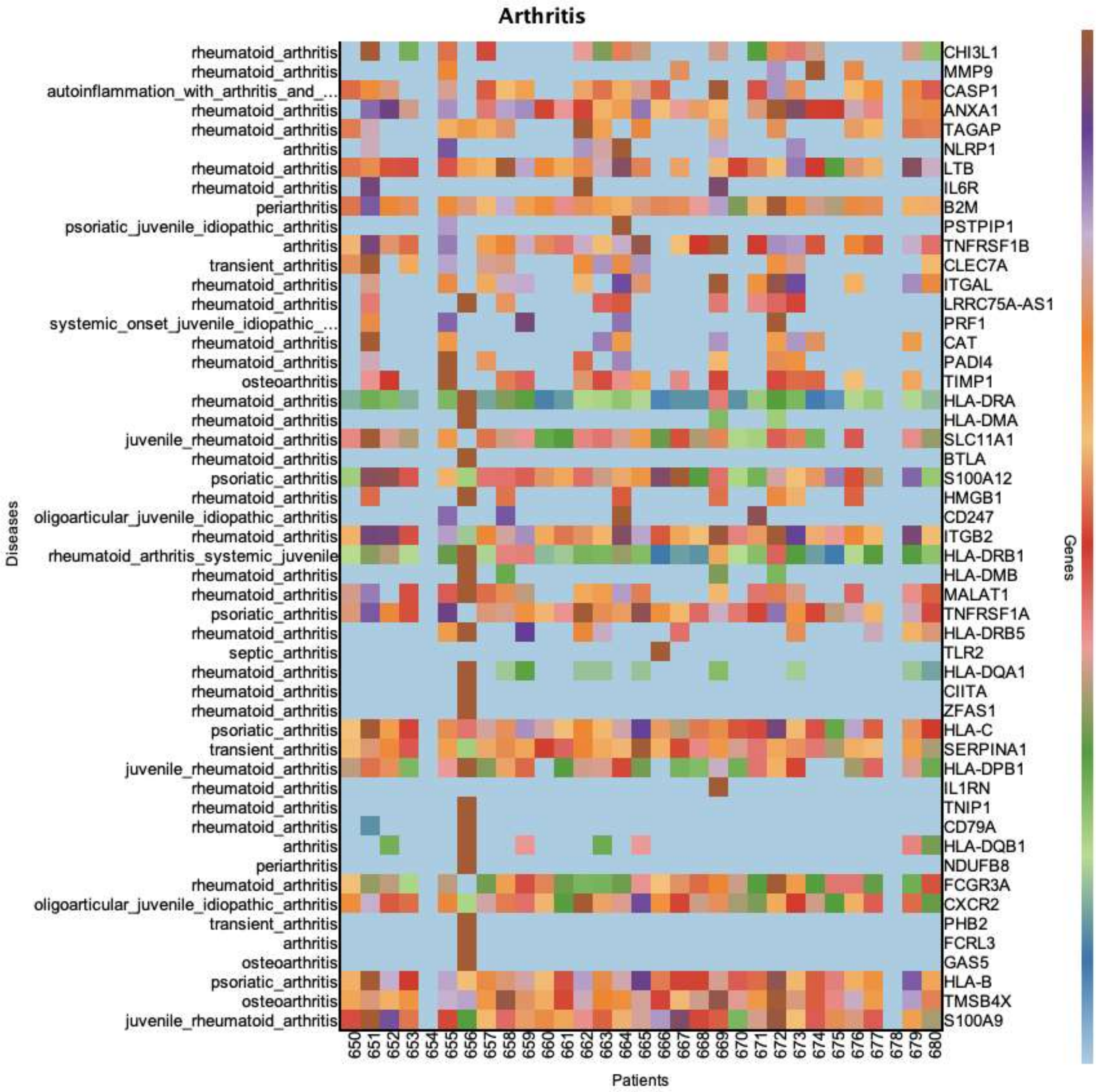

S. Figure 2. Arthritis

GVViZ © Ahmed Lab, Rutgers University, 2021.

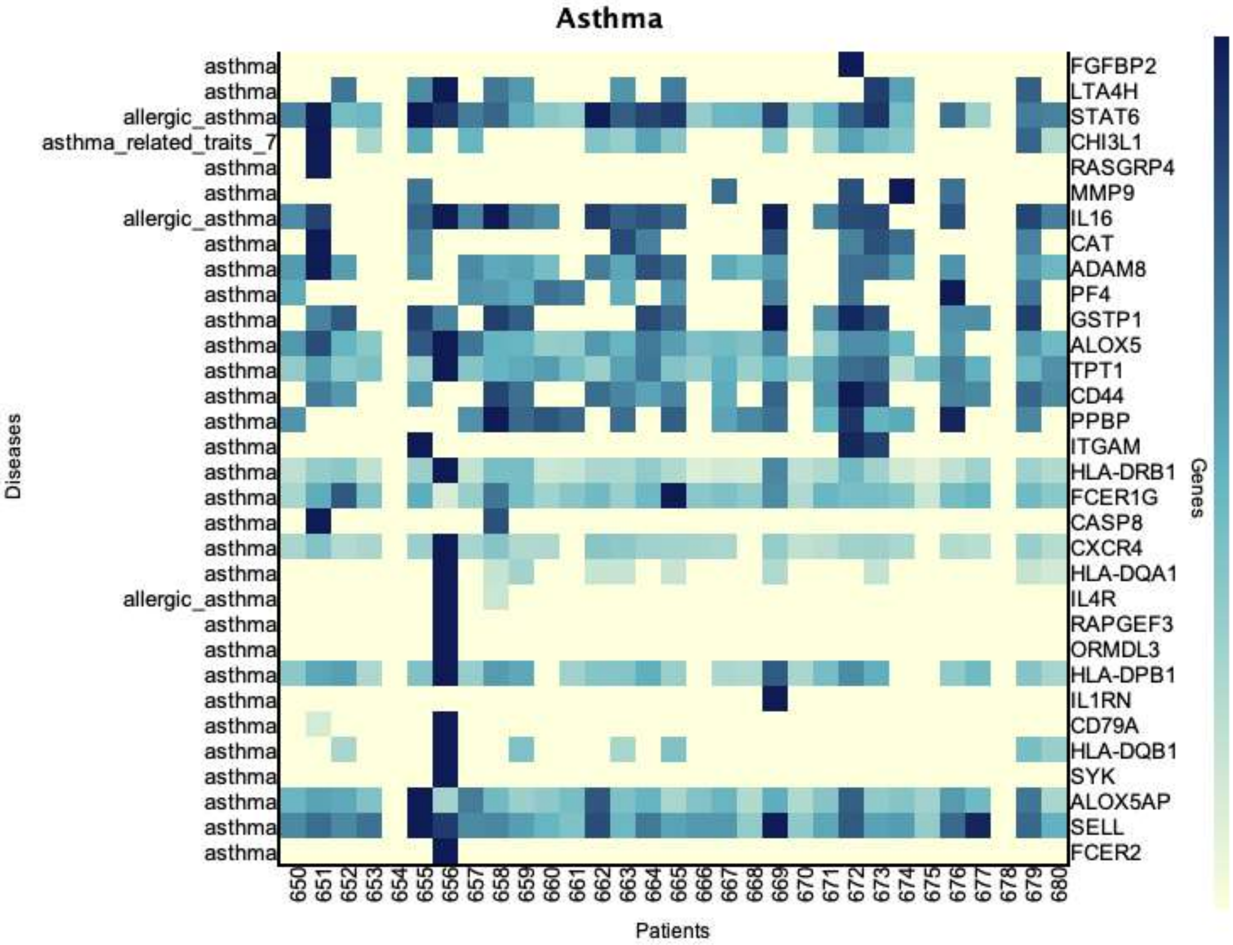

S. Figure 3. Asthma

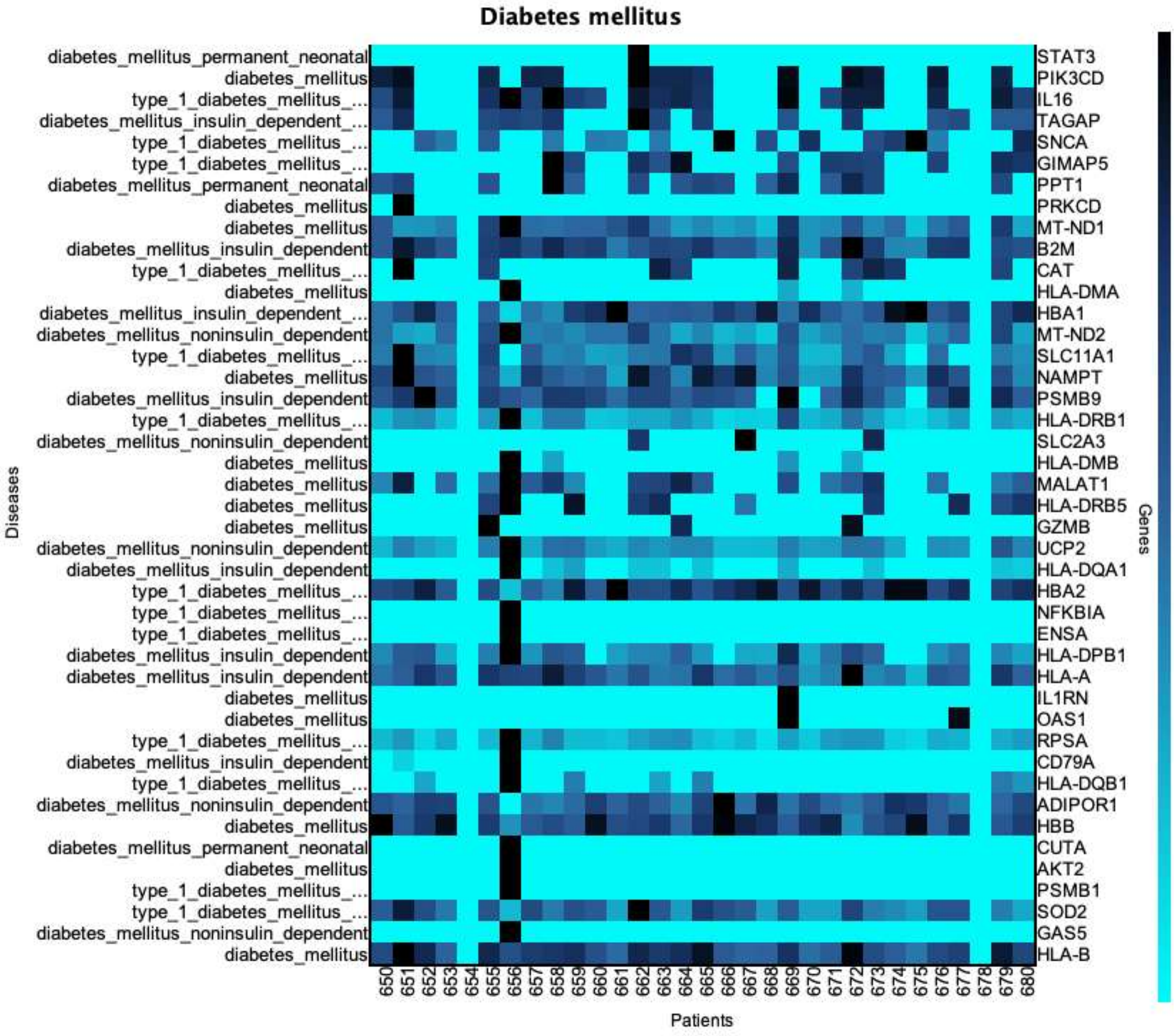

S. Figure 4. Diabetes mellitus

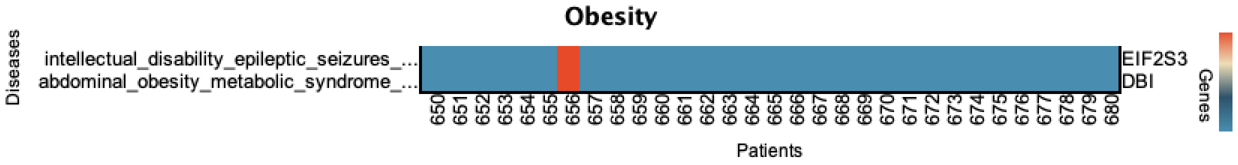

S. Figure 5. Obesity

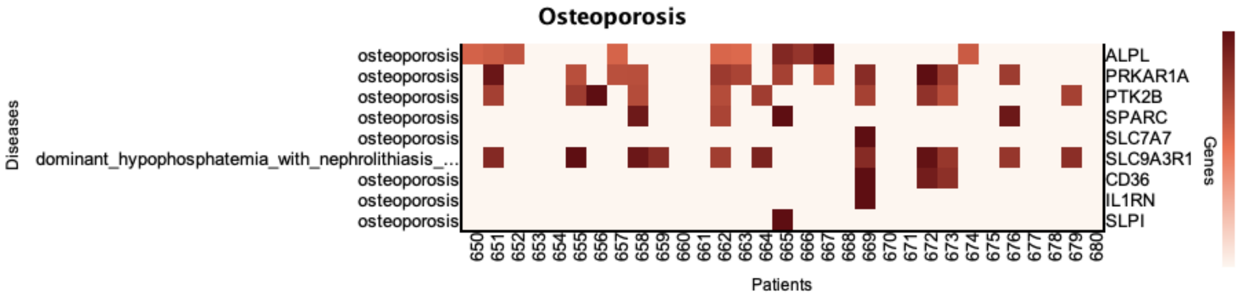

S. Figure 6. Osteoporosis

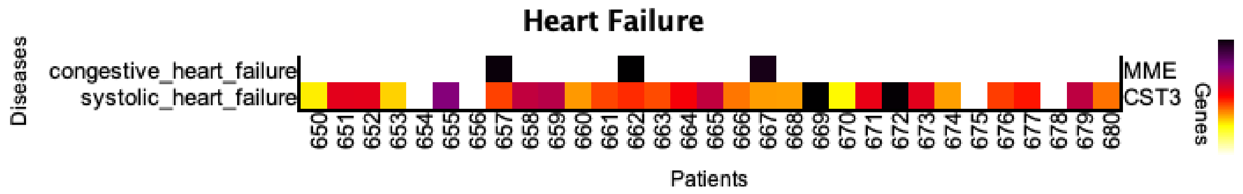

S. Figure 7. Heart failure

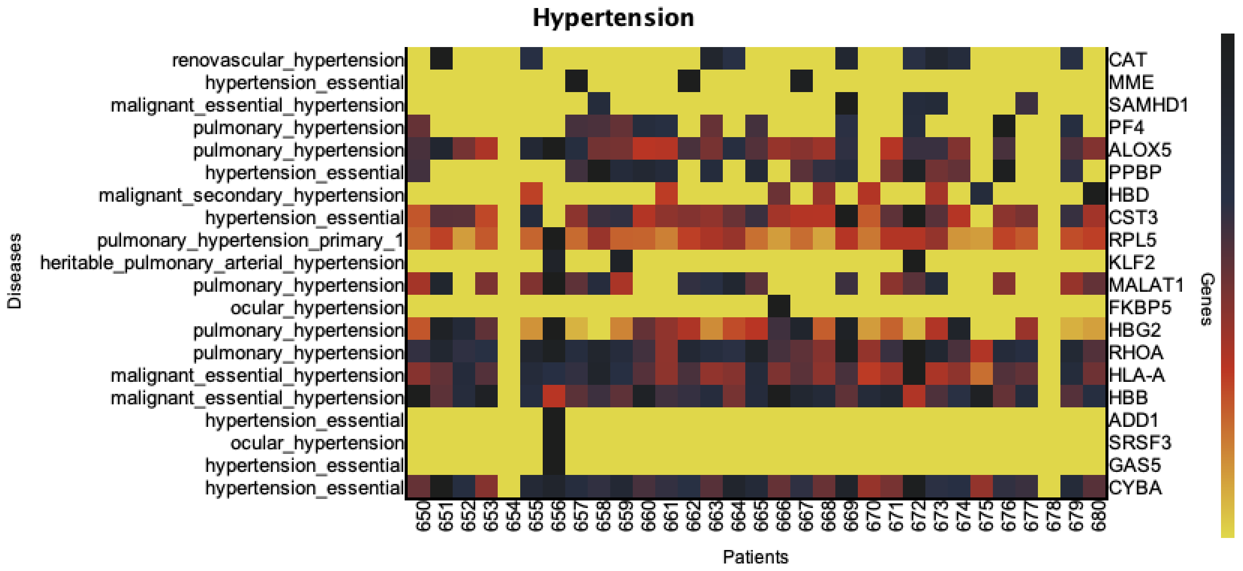

S. Figure 8. Hypertension

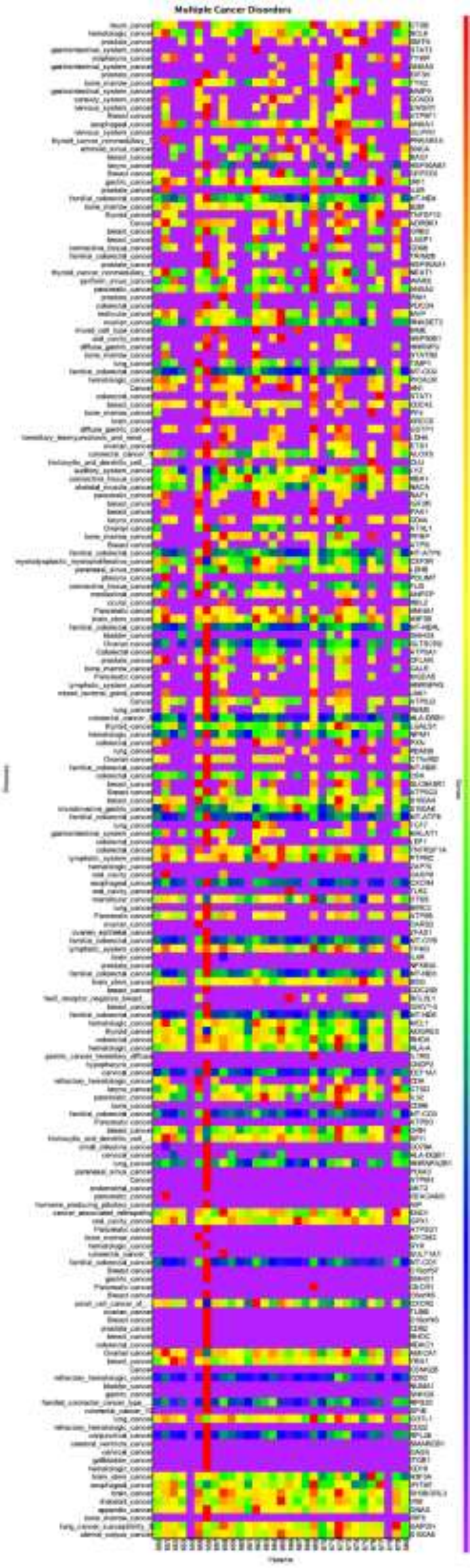

S. Figure 9. Multiple Cancer disorders

GVViZ © Ahmed Lab, Rutgers University, 2021.

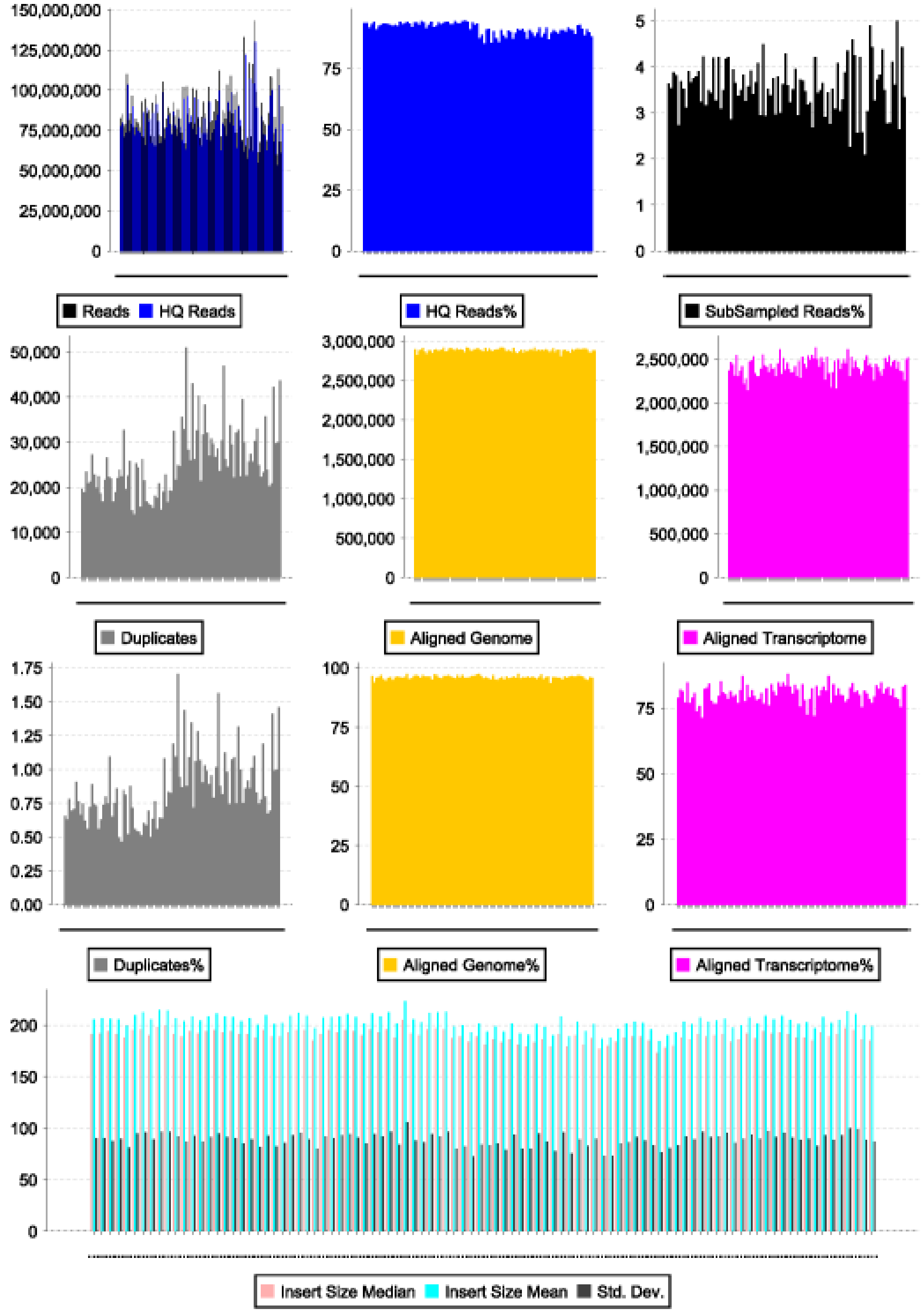

S. Figure 10. QC report of the samples produced using the RNA-seq pipeline.
